# Core–Shell Mn- and Cu-Doped CoFe_2_O_4_@Co_3_O_4_ Hollow Spheres with Dual Adsorption and Catalytic Function

**DOI:** 10.1021/acs.inorgchem.5c04962

**Published:** 2026-01-22

**Authors:** Tetiana Tatarchuk, Wojciech Macyk, Vitaliy Bilovol, Krystian Sokolowski, Marcin Sikora, Kamila Sobańska, Piotr Pietrzyk

**Affiliations:** † Faculty of Chemistry, 37799 Jagiellonian University, Gronostajowa str. 2, 30-387 Kraków, Poland; ‡ Educational and Scientific Center of Materials Science and Nanotechnology, Vasyl Stefanyk Carpathian National University, 76-018 Ivano-Frankivsk, Ukraine; § Jerzy Haber Institute of Catalysis and Surface Chemistry Polish Academy of Sciences, Niezapominajek str. 8, 30-239 Kraków, Poland; ∥ Academic Centre for Materials and Nanotechnology, AGH University of Krakow, Av. Mickiewicza 30, 30-059 Kraków, Poland; ⊥ National Synchrotron Radiation Centre SOLARIS, Jagiellonian University, Czerwone Maki str. 98, 30-392 Kraków, Poland

## Abstract

This work reports the first solvothermal synthesis of Mn- and Cu-doped CoFe_2_O_4_@Co_3_O_4_ hollow spheres. The process leads to the formation of core–shell spinel structures via Ostwald ripening. The resulting materials with surface areas up to 115 m^2^/g were evaluated as bifunctional adsorbent-catalysts for removing Congo Red (CR) dye and oxytetracycline (OTC) from water via combined adsorption and Fenton-like degradation. Mn- and Cu-doping significantly change the morphology of hollow spheres, improving surface charge, redox activity, and pollutant affinity. Mn-doping enhanced the removal efficiency mainly through adsorption, with a minor catalytic contribution. The Mn-3 sample exhibited the highest activity, removing 99% of OTC within 30 min and achieving complete CR elimination within 40 min. XAS and XPS revealed that Co, Mn, and Cu act as redox-active centers, while Fe remains chemically stable. EPR spin-trapping experiments indicated that Cu-3 produces the highest HO^•^ level, whereas Mn-3 is the most effective generator of singlet oxygen. Postreaction XPS confirmed pollutant adsorption, while recyclability tests revealed that CR degradation remains efficient over three cycles, but OTC removal declines, likely due to stronger chemisorption. These results demonstrate the promise of Mn- and Cu-doped cobalt ferrite hollow spheres as multifunctional materials for advanced water treatment applications.

## Graphical abstract

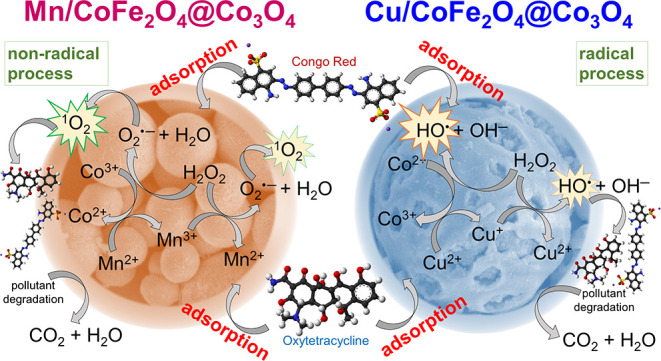

## 1 Introduction

Nowadays, hollow structures with diverse hierarchical architectures have attracted significant interest for both fundamental research and practical applications across a wide range of fields, including catalysis/photocatalysis,^1^ adsorption,^2^ energy storage,^3^ etc. Hollow spheres are tiny, lightweight structures with large internal cavities and tunable pores, providing a large surface area that is ideal for trapping and degrading various pollutants.^4^ Over the past decade, researchers have explored a wide range of hierarchical hollow spheres. They include single-shell metal oxides^5^ and multishell hybrids.^6^ Such structures might be obtained via templated^4^ or self-templated routes.^6^ Templated approaches often yield precisely defined shell thicknesses and void volumes, while self-templated processes, such as Ostwald ripening or the Kirkendall effect, exploit in situ chemical transformations to generate hollow interiors without added templates.^7^ Ostwald ripening, due to the IUPAC definition, is a dissolution of small crystals or sol particles and the redeposition of the dissolved species on the surfaces of larger crystals or sol particles.^7^ Because Ostwald ripening involves complex solid-solution processes, understanding species transport in solution is key to controlled synthesis. It is important to identify chelating ligands that could remove metal ions from crystals, as well as coordination complexes that may redeposit, depending on their chemistry and reactivity.

One synthesis method for obtaining hollow structures is the solvothermal method using glycerol and isopropanol.^8,9^ Such a combination of precursors yields a high-quality architecture with hollow interiors and surface porosity. To date, hollow metal oxide spheres synthesized via solvothermal methods using isopropanol and glycerol as solvents have been studied primarily as electrode materials for batteries and supercapacitors. In particular, it was shown that the yolk–shell structure, formed during solvothermal synthesis with IPA and glycerol, enhanced ion/electron transport, improved electrode stability, and increased surface area for electrolyte access, enhancing electrochemical performance.^10^ Solvothermal reaction, which uses glycerol and isopropanol, has been used for obtaining Fe-glycerate microspheres,^11^ Co_3_O_4_,^12^ Co-glycerate microspheres,^13^ Ce_a_Mn_b_O_*x*_ microspheres,^14^ Fe_3_O_4_,^9^ CuFe_2_O_4_.^1^ However, the potential of hollow structures in environmental catalysis, particularly as Fenton-like catalysts for water purification, remains largely unexplored. Only a limited number of studies have examined the Fenton-catalytic activity of hollow structures obtained by solvothermal synthesis using IPA and glycerol as precursors. For example, Yang et al.^13^ used the Co-glycerate microspheres as an effective catalyst to help break down rhodamine B, a hard-to-remove pollutant, in water using peroxymonosulfate. The same team also used the Fe-glycerate microspheres as a heterogeneous peroxymonosulfate activator to degrade methylene blue.^11^

What really makes spinel hollow spheres exciting for water treatment is their dual role as adsorbent and catalyst when loaded with iron or other transition metals.^15,16^ First, pollutants are attracted to the large surface area of the hollow spheres, both inside and outside. Then, when hydrogen peroxide is added, the metals trigger Fenton-like reactions that produce powerful hydroxyl radicals generated in situ from H_2_O_2_.^1,15^ These radicals rapidly degrade the molecules of organic contaminants into harmless byproducts.^16^ Since pollutants are concentrated precisely where reactive oxygen species are formed, hollow-sphere catalysts can remove toxins quickly, operate under mild conditions, and be reused.^1^ Spinel ferrites, which contain iron and other transition metals, are among the most studied materials in this context.^17^ Spinel ferrites with the general formula MFe_2_O_4_, where M is a transition metal, have attracted significant attention due to their strong magnetic properties, structural stability, and high catalytic activity.^18^ Moreover, the synthesis of spinel ferrites in hollow-sphere form is a relatively new and promising research direction. Additionally, doping ferrites with transition metals such as Mn and Cu can further enhance their redox behavior and surface reactivity.^19,20^ Importantly, designing spinel ferrites with hollow spherical morphology offers several key advantages: (i) a high surface-to-volume ratio that enhances the availability of active sites, (ii) an open pore network that facilitates the mass transfer of reactants and products, and (iii) a low-density structure that supports diffusion-controlled processes such as adsorption and Fenton-like catalytic oxidation. These structural features are especially beneficial for multifunctional systems, where both adsorption capacity and catalytic efficiency are important. Particularly, Ding et al.^1^ reported a magnetic hollow-sphere CuFe_2_O_4_ catalyst rich in oxygen vacancies (HS-CuFe_2_O_4−σ_) that efficiently activates H_2_O_2_ to degrade ciprofloxacin. Zhou et al.^16^ have developed a new, easy one-pot method to make Mn-doped magnetite hollow microspheres that act as an effective Fenton-like catalyst over a wide pH range (4.5–9.5). It was shown that manganese ions were evenly distributed within the magnetite lattice, explaining the structure’s formation via Ostwald ripening. It was found that the catalyst worked through two kinds of active sites: iron centers turned H_2_O_2_ into HO^•^, while manganese centers generated superoxide O_2_^•–^ and hydroperoxyl HOO^•^ radicals, which then formed singlet oxygen ^1^O_2_.^16^

However, there are no reports on the solvothermal synthesis of Mn- and Cu-doped cobalt ferrite@Co_3_O_4_ hollow spheres using isopropanol and glycerol as solvents. Thus, in this study, we report a synthesis and characterization of core–shell Mn- and Cu-doped CoFe_2_O_4_@Co_3_O_4_ hollow spheres. Moreover, we demonstrate how the synergy between the hollow architecture and site-specific cation doping enhances adsorption, directs H_2_O_2_ activation into ROS pathways, and improves the removal of Congo Red and oxytetracycline from water.

## 2 Experimental Section

### 2.1 Chemicals

Cobalt­(II) nitrate hexahydrate Co­(NO_3_)_2_·6H_2_O (CAS 10026–22–9), iron­(III) nitrate nonahydrate Fe­(NO_3_)_3_·9H_2_O (CAS 7782–61–8), copper­(II) nitrate hemi­(pentahydrate) Cu­(NO_3_)_2_·2.5H_2_O (CAS 19004–19–4), and manganese­(II) chloride tetrahydrate MnCl_2_·4H_2_O (CAS 13446–34–9) were purchased from Merck. Sodium hydroxide, 2-propanol, and glycerol were purchased from Chemland (Poland). Congo red C_32_H_22_N_6_Na_2_O_6_S_2_ (CAS 573–58–0) and oxytetracycline hydrochloride C_22_H_24_N_2_O_9_·HCl (CAS 2058–46–0) were purchased from Merck. Hydrogen peroxide (30% solution) (CAS 7722–84–1). Deionized water used in the experiments was sourced using the Hydrolab HLP system. DMPO (CAS 3317–61–1) and TEMP (CAS 768–66–1) were purchased from Merck.

### 2.2 Synthesis of Samples

Cation-substituted cobalt ferrites were synthesized using the solvothermal method. First, the 2-isopropanol (100 mL) and glycerol (30 mL) were mixed for 10 min. Subsequently, stoichiometric amount of metal salts (Co­(NO_3_)_2_·6H_2_O, MnCl_2_·4H_2_O (or Cu­(NO_3_)_2_·2.5H_2_O), and Fe­(NO_3_)_3_·9H_2_O) was added to obtain the final composition of Co_1–*x*_Mn_*x*_Fe_2_O_4_ or Co_1–*x*_Cu_*x*_Fe_2_O_4_ (*x* = 0, 0.1, 0.3), and the mixture underwent magnetic stirring for 1 h. The formed transparent solution was then transferred to the Teflon autoclave and heated at 180 °C for 12 h. After natural cooling, the resulting precipitates were centrifuged at 3000 rpm, washed several times with deionized water and ethanol, and finally dried at 60 °C for 5 h. The obtained precursors were annealed at 300 and 400 °C for 3 h in air (the heating rate was 2 °C/min). The obtained powders were labeled as CFO, Mn-1, Mn-3, Cu-1, and Cu-3.

### 2.3 Characterizations and Analysis Methods

The samples were characterized using a wide range of complementary techniques to study their structure, surface chemistry, and electronic properties, as described in detail in the Supporting Information (Text S1).

### 2.4 Fenton-like Oxidation of Pollutants

Catalytic wet peroxide oxidation experiments were carried out using synthesized samples as Fenton-like catalysts, hydrogen peroxide as the oxidant, and model organic pollutants namely Congo Red (CR) dye and the antibiotic oxytetracycline (OTC). All reactions were conducted in a total solution volume of 25 mL, containing 20 mg/L of the pollutant (either CR or OTC), 10 mM H_2_O_2_, and 1 g/L of catalyst. The initial rate of CR or OTC degradation was determined by monitoring the decrease in pollutant concentration over time. Reactions were performed under constant temperature and stirring conditions. Aliquots were withdrawn at fixed time intervals, and the residual concentration of CR or OTC was measured using UV–vis spectroscopy at their respective maximum absorption wavelengths (498 nm for CR and 354 nm for OTC). The initial reaction rate *r*_0_ was calculated from the slope of the linear part of the *C*_τ_/*C*_0_ vs time plot, where *C*_0_ is the initial pollutant concentration, and *C*_τ_ is the concentration at time τ (in minutes). To further investigate the interaction between the pollutant and the catalyst surface, additional experiments were performed in two sequential stages: an initial adsorption phase without H_2_O_2_ (60 min, with monitoring of adsorption kinetics), followed by the addition of H_2_O_2_ to initiate catalytic degradation of the remaining pollutant. As before, aliquots were withdrawn at regular intervals, and residual concentrations were measured by UV–vis spectroscopy, with initial degradation rates calculated accordingly. Additionally, the catalytic activity of the most effective catalyst was evaluated for the simultaneous degradation of a CR–OTC mixture, with each pollutant at 10 mg/L (total pollutant concentration: 20 mg/L). Degradation progress was monitored via UV–vis spectroscopy, considering the distinct absorption maxima of the two pollutants (498 nm for CR and 345 nm for OTC).

### 2.5 Cycling Experiments

The recycling performance of the catalysts was also examined. The catalyst used in the degradation experiments was collected using a neodymium magnet and then rinsed with water. Finally, the wet catalyst was used for subsequent experiments.

### 2.6 Radical Identification

To identify reactive oxygen species in the liquid phase, EPR and spin-trapping techniques were used. Measurements were performed on a MiniScope MS400 spectrometer (Magnettech) operating in the X band (∼9.5 GHz). The spectra were recorded at room temperature with a magnetic field modulation amplitude of 0.2 mT. A DMPO (5,5-dimethyl-1-pyrroline N-oxide) spin trap was used to detect hydroxyl radicals, while in the case of singlet oxygen, the reaction with TEMP (2,2,6,6-tetramethylpiperidine) was used. The solutions for measurements were prepared as follows: to 0.5 mg of catalyst suspension, 0.1 mL of 0.1 mol/dm^3^ hydrogen peroxide solution, and 1 mL of 20 mmol/dm^3^ DMPO or TEMP. The mixture prepared in this way was transferred to a glass capillary and placed in the spectrometer cavity.

## 3 Results and Discussion

### 3.1 Morphological and Structural Characterization

The solvothermal method has been chosen to synthesize Mn- and Cu-doped cobalt ferrites because changing the solvent molecules allows control over the formation of metal oxides with a designed morphology.^9^ At the first step, the as-prepared samples (precursors), consisting of numerous microspheres with 1–3 μm in diameter, have been obtained (Figure 1). The precursors were finally annealed at 300 and 400 °C with a heating rate of 2 °C/min, which resulted in the spinel structure formation (Figure 2a). It is worth noting that the mixture of glycerol, isopropanol (IPA), and metal ions leads to the formation of solid spheres, as a consequence of coordination of isopropanol to metal ions (Co^2+^, Fe^3+^, Mn^2+^, and Cu^2+^) under pressure during the solvothermal process.^21^ These solid spheres are not thermodynamically stable and transform into stable glycerolates,^9,22^ which are coordination compounds with Co^2+^ or Fe^3+^ ions chelated by glycerol molecules (acting as multidentate ligands). They can form core–shell structures, where CoFe-IPA, CoMnFe-IPA, and CoCuFe-IPA constitute cores, while metal-glycerolates form the shells. Given that the process is carried out for 12 h at 180 °C, it results in the transformation of the metal-IPA core and glycerolate shell into spinel structures, with a tendency to form a space between the core and the shell. SEM analysis shows that the morphology of the precursors differs somewhat from that of the annealed samples. The as-prepared CFO sample contains flower-like species with a diameter of 2–3 μm and smooth spherical species (Figure 1a–e). Ultrathin nanosheets form each flower with an average thickness of around 60 nm. Mn-containing samples exhibit microsphere morphology but lack the flower-like architecture (Figure 1b,c). Additionally, greater the Mn content, the smoother the spheres’ surface. Cu-containing precursors also form spheres, which become increasingly damaged as the copper content increases (Figure 1d,e).

**Figure 1:**
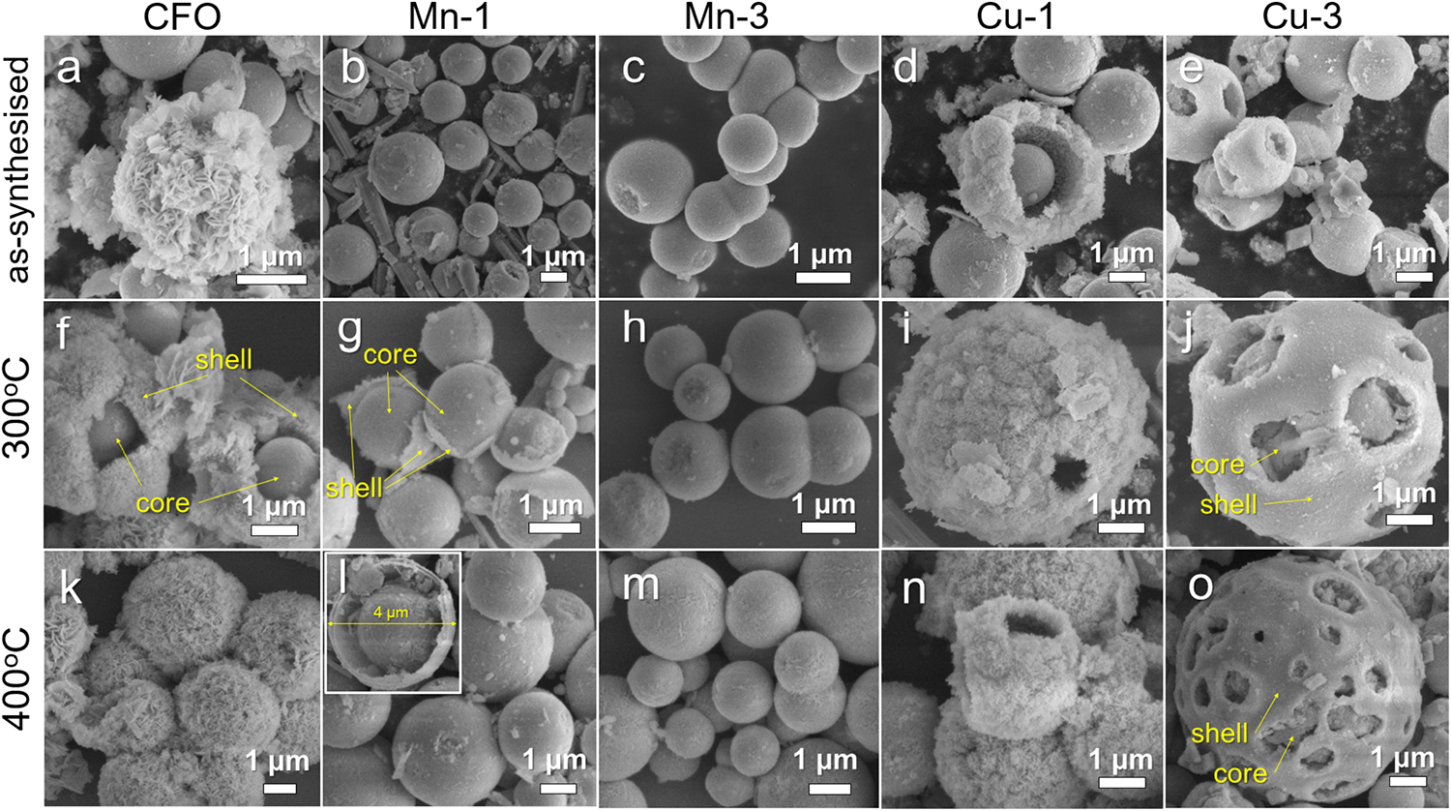
SEM pictures of various morphologies of (a–e) as-synthesized precursors and samples annealed at (f–j) 300 °C and (k–o) 400 °C.

**Figure 2:**
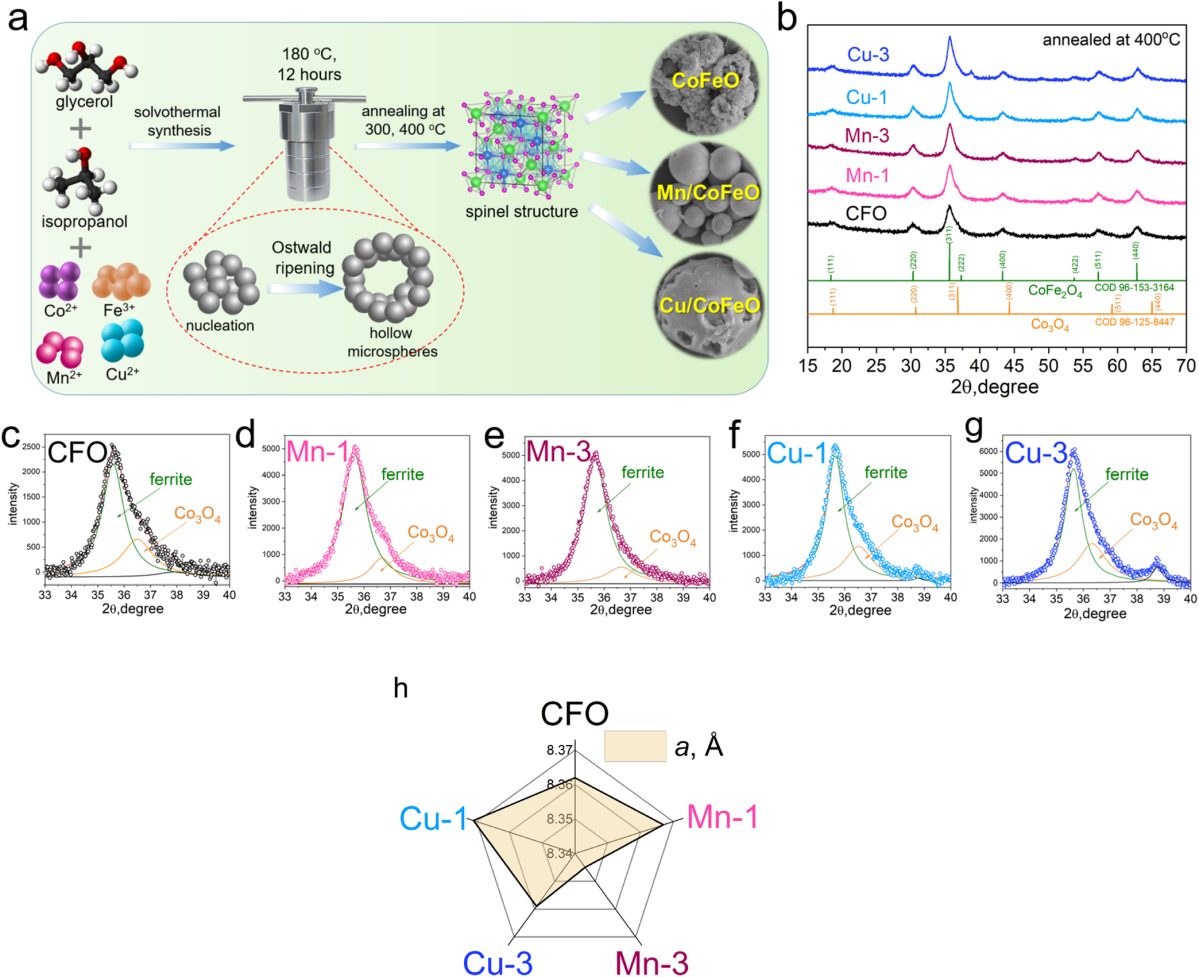
(a) Schematic illustration of the solvothermal synthesis of Mn- and Cu-containing cobalt ferrite hollow spheres. (b) XRD patterns of the samples annealed at 400 °C. (c–g) The deconvolution of the main (311) peak. (h) The lattice parameter of the ferrites.

After annealing at 300 °C, the morphology of the precursor particles changed (Figure 1f–j). The clearly visible core–shell structure is observed for the CFO, Mn-1, and Cu-3 samples. The external diameters of hollow-sphere particles range from 2 to 4 μm. The thickness of ultrathin nanosheets for the CFO sample decreased to 30–40 nm after annealing. Calcination at 400 °C does not alter the morphology of the spheres significantly (Figure 1k–o). Similar observations were reported in refs 12 and 23 . The nanosheets of the flower-like CFO material can also withstand heating. The presence of nanosheets ensures a highly porous structure, thereby providing a high specific surface area (see Section 3.5). A more porous surface is observed for the Mn-3 sample annealed at 400 °C (Figure 1m). Under these conditions, the surface of each sphere becomes rougher, in contrast to the corresponding samples obtained at 300 °C. The structure of the Mn-1 sample, annealed at 400 °C, exhibits a core–shell arrangement, featuring a conspicuous gap between the solid internal core and the outer shell (Figure 1l). The introduction of copper ions also alters the particle morphology (Figure 1n,o). For example, in the Cu-1 sample, the ability of particles to form flower-like sheets is still partially retained. However, this morphology disappears in the Cu-3 sample. This change is most likely due to the lower concentration of cobalt ions in the system. As reported in previous studies,^24,25^ cobalt ions play a key role in promoting the formation of flower-like sheet morphology. The presence of copper ions promotes the formation of particles with a smoother surface. The increase in Cu content also results in cracks on the particle surface (Figure 1e,j,o). A well-developed “core–shell” type structure in the micron-sized particles was formed after annealing at 300 and 400 °C (Figure 1f,g,j,o).

It should be noted that the primary role in the formation of spinel microspheres is attributed to a phenomenon known as the “Ostwald ripening process”.^26,27^ This process involves the dissolution of smaller crystals and the subsequent redeposition of the dissolved material onto the surfaces of larger crystals.^27,28^ Such behavior may facilitate the growth and development of core–shell crystal structures over time.^29^ Dong et al. indicated that the formation of initial Co-glycerolate solid spheres is subsequently followed by the growth of Fe-glycerolate on the surface of the Co-glycerolate spheres.^27^ In our case, Ostwald ripening occurs due to differences in the solubility of metal glycerolates in organic solvents, such as isopropanol and glycerol.^30^ The process begins with nucleation; under the influence of glycerol and isopropanol, metal salts thermally convert into glycerolates. Literature suggests that glycerolate spheres can be produced after a relatively short heating period, typically up to 6 h, from dilute salt solutions.^27^ In our study, we utilized saturated solutions and did not confirm the formation of glycerolates using XRD (Figure 2b).

It is possible that this stage was intermediate, and that heating for 12 h led to the development of a mixture of prespinel phases, including oxides, oxyhydroxides, and spinel nuclei. This suggests that the solutions reached supersaturation, causing some glycerolates to dissolve and then redeposit onto particles of other glycerolates. Additionally, external particles formed a porous shell of flower-like nanosheets (Figure 1). This redistribution process is crucial, as it provides the foundation for the subsequent growth of Mn- and Cu-containing spinel architectures with a hollow sphere morphology. The phase transition of the internal microspheres may be influenced by surface activity, thereby enhancing ion mobility required for the formation and growth of spinel ferrite.

The rearrangement of initially formed particles into spinel microspheres likely requires minimal energy, which empowers this transformation. Ultimately, the core–shell spinel structures are formed, where the core consists of ferrite spherical particles and the shell is made of porous ferrite nanosheets (see Figure 3). The proposed mechanism suggests that smooth microspheres crystallize into core–shell structures through a multistep reaction involving an IPA/glycerol mixture and a dissolution-recrystallization process, which leads to the nucleation and growth of spinel ferrite nanoparticles. Importantly, in this case, glycerol is essential in creating a viscous medium that is beneficial for nanoparticle synthesis.^31^

**Figure 3:**
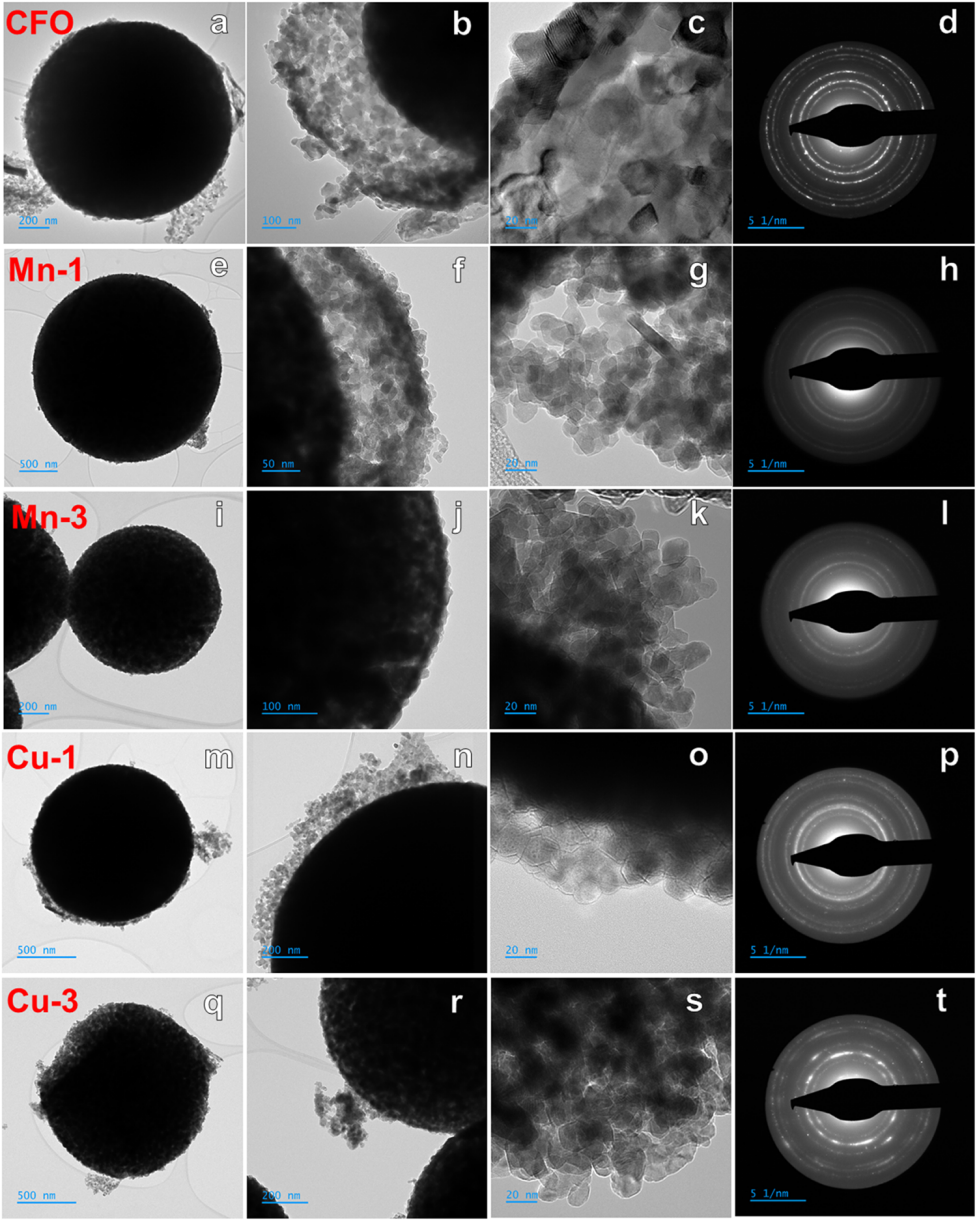
TEM images of Mn- and Cu-substituted cobalt ferrites, annealed at 400 °C: (a–c) CFO, (e–g) Mn-1, (i–k) Mn-3, (m–o) Cu-1 and (q–s) Cu-3 samples. SAED patterns for (d) CFO, (h) Mn-1, (l) Mn-3, (p) Cu-1 and (t) Cu-3 samples.

The XRD patterns of powders, synthesized by the solvothermal method, are depicted in Figures S1 and 2b. No broad diffraction peak at 10–12° is observed, which is typically associated with the formation of the metal-glycerolate phase in the glycerol medium.^27^ Diffraction peaks between 20° and 50° can be attributed to FeOOH (JPCDS no. 81–0462), indicating the presence of low crystallinity FeOOH in the as-prepared samples.^27^ The samples annealed at 300 and 400 °C reveal the formation of the spinel phase (Figures S1b and 2b). It is noteworthy that two distinct spinel phases are formed: cobalt ferrite CoFe_2_O_4_ and cobalt oxide Co_3_O_4_ (Table S1). Reflections indexed to CoFe_2_O_4_ (COD 96–153–3164) correspond to the (111), (220), (311), (222), (400), (422), (511), and (440) crystal planes. The reflections assigned to the Co_3_O_4_ (COD 96–125–8447) correspond to the (111), (220), (311), (400), (511), (440) crystal planes. Figure 2c–g display the magnified (311) reflection within the 2θ range of 33–40°. The most intensive peak, centered at ∼36°, exhibits an asymmetric profile with a noticeable shoulder on the higher-angle side, indicating the coexistence of the Co_3_O_4_ phase in the samples.^32^ The presence of Co_3_O_4_ in the samples can be attributed to surface cobalt enrichment and subsequent oxidation during annealing in an oxygen-containing atmosphere, resulting in a Co_3_O_4_ shell over the ferrite core. This core–shell structure arises from the diffusion of Fe^3+^ into the interior and preferential oxidation of surface Co^2+^ to Co^3+^ (compare to the XPS and XAS data below). All samples exhibit high crystallinity, as evidenced by sharp, well-defined diffraction peaks. An increase in Mn or Cu content results in a higher intensity of the (311) peak, suggesting enhanced crystallization of spinel nuclei in the presence of these dopant ions. Additionally, the Cu-1 sample contains about 2%, while the Cu-3 sample contains about 5% of CuO.

Figure 2h shows the variation in the lattice parameter of ferrites. For the pristine CoFe_2_O_4_ sample, the lattice parameter is 8.362 Å. The initial introduction of Mn^2+^ and Cu^2+^ ions leads to an increase, reaching 8.367 Å for the Mn-1 sample and 8.371 Å for the Cu-1 sample. This increase can be mainly attributed to the larger ionic radius of Mn^2+^ (0.83 Å) compared to Co^2+^ (0.745 Å)^33^ as it is incorporated into the lattice, leading to lattice expansion and a lattice strain reduction. For the Cu-1 sample, although Cu^2+^ ions are slightly smaller than Co^2+^ (*r*(Cu^2+^) = 0.73 Å), their strong preference to octahedral sites, combined with Jahn–Teller distortion^34^ and the resulting cation redistribution, can expand the lattice, leading to the observed increase in *a* parameter. Further substitution of Mn (in the Mn-3 sample) leads to a slight decrease in the lattice parameter to 8.345 Å. This decrease may be explained by potential cation redistribution between the A- and B-sites, the partial oxidation of Mn^2+^ to the smaller Mn^3+^ species (0.645 Å), or a rise in structural disorder compensating for the earlier expansion.^35^ As the Cu content increases (in the Cu-3 sample), the lattice parameter also decreases significantly to 8.359 Å. In this case, this trend aligns with the smaller size of Cu^2+^ and the stabilization of the Jahn–Teller effect, resulting in a net reduction in unit cell volume. In addition, the presence of Cu^+^ species is possible in the Co–Cu ferrite samples due to the use of isopropanol and glycerol, which act as mild reducing agents. The reducing properties of these organic reagents likely facilitated the partial reduction of Cu^2+^ to Cu^+^ during the early stages of thermal treatment. The retention of Cu^+^ after calcination suggests that localized reducing conditions may have persisted or that Cu^+^ was stabilized within the spinel structure, potentially at surface or defect sites.^36^ The coexistence of Cu^+^ and Cu^2+^ suggests a mixed-valence state that may contribute to cation redistribution and overall lattice contraction. Cu^+^ with its smaller ionic radius (0.60 Å)^36^ can lead to a more compact spinel lattice, thereby explaining the observed reduction in lattice parameter with increased Cu doping. This mixed-valence state (as indicated by the XAS and XPS data below) could enhance electron-transfer processes, contributing to improved Fenton-like catalytic activity of the catalysts in the degradation of organic pollutants. The chemical composition of the studied samples was examined using EDS and the results are presented in Text S2 and Figure S2.

The morphology of the samples annealed at 400 °C was also examined by TEM. Figure 3 illustrates the formation of hollow spheres 1–2 μm in diameter, with surfaces densely packed with small nanoparticles.

Figure 3a,e,i,m,q clearly show well-defined spherical particles with hollow interiors, indicative of a core–shell architecture. The shell structure consists of closely packed nanocrystallites, each measuring approximately from 10 to 20 nm, which collectively form porous walls, as shown in Figure 3b,f,n. The samples Mn-3 and Cu-3 exhibit thinner shell structures. The unique hollow morphology of the Mn- and Cu-doped spheres, combined with the nanoscale crystallinity and porosity of the shell, suggests that these materials possess structural characteristics favorable for Fenton-like heterogeneous catalysis (Figure S3a–c). These include a high surface-to-volume ratio, enhanced diffusion of reactants and products, and increased accessibility of active sites.^15^ Furthermore, the SAED patterns confirm the polycrystalline nature of the spinel samples, as demonstrated by multiple well-defined lattice planes. The presence of lattice fringes in the HR-TEM images further substantiates the crystalline structure of all samples, as depicted in Figure 3d,h,l,p,t. For example, the HR-TEM image displaying a well-ordered atomic lattice in the Mn-3 sample is shown in Figure S3d.

FTIR spectroscopy has been used to identify functional groups in the samples’ structures. As shown in Figure S4a, the spectra of the as-synthesized samples contain multiple peaks and differ significantly from the spectra of annealed ones (Figure S4b,c). The detailed analysis is presented in the Supporting Information (Text S3). The FTIR spectra exhibit strong low- and high-frequency bands in the 350–600 cm^–1^ region, representing the stretching vibrations of the bonds between metal ions placed in octa- or tetra-positions of the spinel structure and oxygen ions.^37^ The observed spectral shifts in the annealed samples provide compelling evidence of the integration of Cu and Mn ions into the cobalt spinel structure. They can be attributed to the substitution of Co by Mn or Cu, which results in lattice strain and a crystal-field effect due to the different ionic radii. The spectra demonstrate that the intensity of tetrahedral mode vibrations is higher than that of octahedral ones. The reason is that the distance between A-metal and oxygen is shorter than between B-metal and oxygen.

### 3.2 Electronic Structure from X-ray Absorption Spectroscopy (XAS)

Figure 4a–d show XAS of the ferrite samples annealed at 400 °C. As observed in the XRD analysis, the X-ray absorption spectra at the Co edge contain contributions from Co atoms belonging to two distinct phases, namely ferrite and Co_3_O_4_, making their interpretation nontrivial. From the XAS collected at Co L_3_-edge (Figure 4a), a variation in the relative intensity of the peaks at about 778 and 780 eV is evident. According to the literature, the latter feature is primarily attributed to Co^3+^ ions in octahedral coordination.^38^ Thus, the XAS shape confirms the presence of Co^3+^ originating from the Co_3_O_4_ identified by XRD, and the observed peak intensity evolution is consistent with its relative abundance in the samples. The X-ray absorption spectra at the Fe edge (Figure 4b) provide information exclusively on the Fe atoms within the cobalt ferrite phase. Therefore, these spectra can be reliably used to analyze the distribution of iron between the octahedral (O_h_) and tetrahedral (*T*_d_) sublattices. The L_2_ edge was selected for this analysis, as this region of the spectrum is susceptible to site occupancy in both coordination environments.^39^ We normalized the signal to the maximum intensity of the absorption line at the L_2_ edge (Figure S5). The ratio *I*_1_/*I*_2_, where *I*_1_ and *I*_2_ represent the intensities of the lower and higher energy peaks in the L_2_ multiplet, respectively, varies between the samples. This variation indicates differences in the occupancy of the O_h_ (*I*_2_) and *T*_d_ (*I*_1_) sites by Fe atoms across the sample sets. Focusing on the samples with the highest doping level, the *I*_1_/*I*_2_ ratio decreases in spinels, where Fe occupancy at tetrahedral sites is reduced, and increases as this occupancy increases. Regarding the inversion degree parameter γ,^40^ this observation has a clear qualitative implication. Since γ reflects the number of Fe atoms occupying *T*_d_ sites, its value is lower for the Cu-series and higher for the Mn-series relative to the undoped sample.

**Figure 4:**
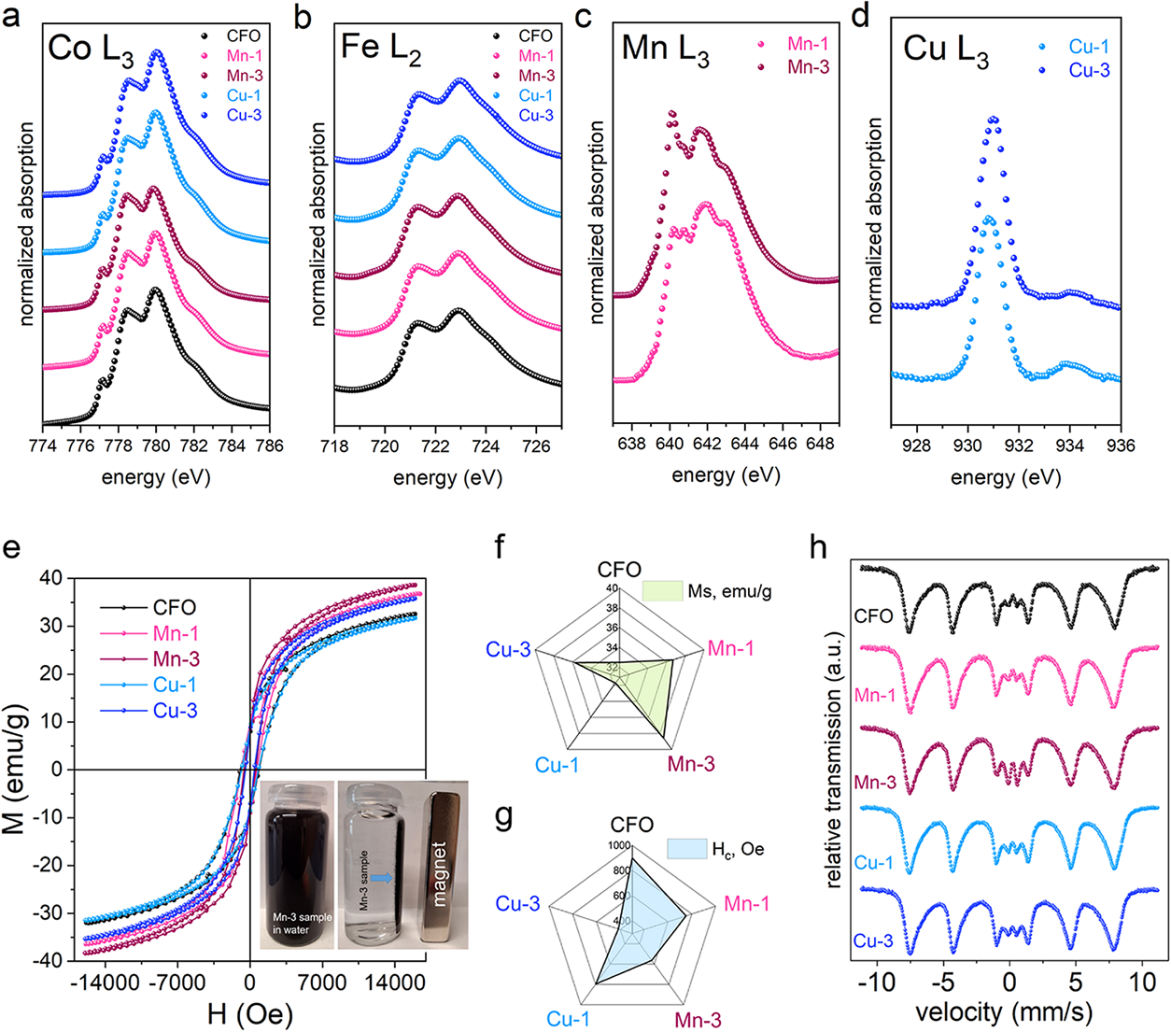
XAS spectra of the ferrites annealed at 400 °C: (a) at the Co L_3_-edge normalized to the intensity of the peak at 778.5 eV of all the samples; (b) at the Fe L_2_-edge normalized to the intensity of the peak at 723 eV of all the samples; (c) at the Mn L_3_-edge normalized to the maximum intensity; (d) the L_3_-edge of Cu. (e) M vs H curves at room temperature (inset: the demonstration of magnetic properties of the Mn-3 sample in aqueous solution). The values of the (f) magnetization (at 1.6 T) and (g) coercivity obtained from the hysteresis loops for all samples. (h) Room temperature Mössbauer spectra of the samples.

The peak at approximately 640 eV in the Mn L_3_-edge absorption is commonly attributed to Mn in the 2+ oxidation state.^41^ The broader peak near 642 eV is typically associated with Mn^3+^, while the feature around 643 eV is characteristic of Mn^4+^ ions.^42^ The Mn spectra were deconvoluted using a linear combination of the reference XAS shapes characteristic of Mn^2+^, Mn^3+^, and Mn^4+^ – constructed using principal component analysis. The resulting quantified compositions were 46% Mn^2+^/43% Mn^3+^/11% Mn^4+^ and 28% Mn^2+^/50% Mn^3+^/22% Mn^4+^ for the Mn-1 and Mn-3 samples, respectively. These results confirm that both samples contain Mn ions in multiple oxidation states with differing proportions. A trend is observed in which an increase in Mn^2+^ content corresponds to a decrease in Mn^4+^ content, and vice versa. Furthermore, it can be inferred that the amount of Co^3+^ ions decreases with increasing total Mn loading.

The Cu L_3_-edge absorption spectra exhibit a characteristic doublet feature (Figure 4d) with an energy separation of approximately 3.0 eV. The dominant peak corresponds to Cu^2+^ ions in the 3d^9^ configuration, featuring a single unoccupied *d*-state.^38^ The weaker, secondary peak is attributed to Cu^+^ ions,^38^ which possess a fully occupied 3d shell and are therefore nonmagnetic. According to the XRD results, in addition to cobalt ferrite and Co_3_O_4_, a spurious CuO phase, paramagnetic at room temperature, is also present. SEM/EDS reveals that Cu is distributed throughout the sample, with some regions exhibiting higher concentrations (Figure S2l). These Cu-rich regions are attributed to the presence of copper in the CuO phase. Accordingly, the Cu^2+^ ions detected in the XAS measurements are associated with this CuO phase, where Cu atoms adopt a planar tetrahedral coordination.^43^ Conversely, the Cu-poor regions are linked to Cu^+^ ions incorporated into the cobalt ferrite phase. These observations suggest that Cu atoms tend to segregate outside the spinel structure. Notably, as the copper content increases, the intensity of the 780 eV feature, attributed to Co^3+^, in the CoCuFeO sample series (Figure 4a) also becomes more pronounced.

While XAS provides detailed information on oxidation states and local coordination, DRS measurements were performed to determine the band gap energies of the synthesized ferrite samples and to estimate the influence of Mn and Cu doping on the electronic structure. The reduction in the optical band gap upon substituting Co ions with Mn or Cu ions in cobalt ferrite is observed, which is attributed to changes in the electronic structure, lattice distortion, and increased delocalization of charge carriers. Taking into account the electronic configuration of the substituting ions (e.g., 3d^7^ for Co^2+^, 3d^5^ for Mn^2+^, and 3d^9^ for Cu^2+^), the replacement of Co^2+^ ions with Mn or Cu introduces new energy states in the d-orbitals, which results in the narrowing of the band gap. These dopants modify the crystal-field environment, affecting the M–O bond lengths and angles and facilitating lower-energy electronic transitions of possible photoactivity relevance. The detailed DRS analysis is presented in Supporting Information (Text S4 and Figure S6).

### 3.3 Magnetic Properties

Figure 4e presents the results of magnetization measurements as a function of applied magnetic field for all samples, conducted at room temperature. The presence of magnetic properties is a key advantage of spinel-based adsorbents and catalysts, as it enables their easy separation from aqueous solutions using an external magnet (Figure 4e (inset)). As shown, magnetization increases with Mn addition, reaching a maximum in the Mn-3 sample (Figure 4f). Similar effects of Mn on magnetization have been reported previously.^44^ Conversely, the coercive field value (H_c_) decreases with Mn doping (Figure 4g). The magnetization of a ferrimagnet can be explained using the Néel model.^45^ According to this model, the net magnetization of a spinel arises from the vector sum of the collinear magnetic moments on two oppositely oriented sublattices: the tetrahedral (A) and octahedral (B) sites. As observed from the hysteresis loops of the Cu-containing samples (Figure 4e), there is an initial slight decrease in magnetization in the Cu-1 sample, followed by an increase in the Cu-3 sample. The coercivity exhibits a similar nonlinear trend: it increases slightly in the Cu-1 sample, then decreases sharply in the Cu-3 sample. Therefore, in the Cu-1 sample, the substitution of Co^2+^ (∼3 μ_B_) at the O_h_ sites with nonmagnetic Cu^+^ results in a reduction of the magnetic moment of the O_h_ sublattice, thereby decreasing the overall magnetization of the sample. In contrast, for the Cu-3 sample, the replacement of Co^2+^ with nonmagnetic Cu^+^ at the *T*_d_ sites reduces the magnetic moment of the *T*_d_ sublattice, which in turn leads to an increase in the net magnetization. Since coercivity is generally inversely proportional to magnetization for single-domain particles (H_c_ ∼ 1/M_S_), it decreases as the magnetization increases.^46^ These observations suggest that the amount of the dopant element (e.g., Cu) governs the site-specific substitution mechanism.

Assuming the magnetic moments of the Fe^3+^ and Co^2+^ ions are 5 μ_B_ and 3 μ_B_, respectively, we consider the magnetic moments of Mn^2+^, Mn^3+^, and Mn^4+^ ions to be approximately 5 μ_B_, 4 μ_B_, and 3 μ_B_, respectively. For example, substituting a Co^2+^ ion with either a Mn^2+^ or a Mn^3+^ ion at the octahedral sites can increase magnetization in the Mn-1 sample relative to the undoped sample. A further increase in Mn doping continues to enhance magnetization, as explained by the same reasoning. Similar behavior in the Co_1–*x*_Mn_*x*_Fe_2_O_4_ system (for *x* ≤ 0.3) has been reported in ref 44. XAS measurements confirmed the presence of Mn in multiple valence states. Based on the observed coercivity trend, we infer that Mn atoms preferentially occupy octahedral sites, replacing Co^2+^ ions, which are known to be the primary contributors to magnetic anisotropy in cobalt ferrites.^47^ Given that the higher concentration of Mn^3+^ (4 μ_B_) is in the Mn-3 sample, it is plausible that the observed increase in magnetization within this series arises from Mn^3+^ ions substituting Co^2+^ ions at octahedral positions. Additionally, Mn ions in other valence states may also occupy tetrahedral sites, potentially replacing Co^2+^ ions and further influencing the magnetic properties.

### 3.4 Iron State from ^57^Fe Mössbauer Spectroscopy

Figure 4h shows the raw Mössbauer spectra at room temperature for the samples annealed at 400 °C. The overall feature is that all spectra exhibit a similar profile. A common characteristic is the presence of distributed Zeeman splitting, with comparable separation between the outermost absorption lines and a central doublet of varying intensity. This pattern is consistent with a system of iron oxide particles of different sizes. The larger particles are nearly magnetically blocked, while the smaller particles remain magnetically relaxed at room temperature, likely residing in the superparamagnetic regime. Similar effects in the same system have been reported in the literature.^48^ Figure S7 illustrates an example of the spectrum fitting for one of the samples (the Mn-3 sample), using magnetic hyperfine field distribution curves. The spectra of the other samples, which were fitted with the same model, are not shown because they are similar. Based on a combination of XRD, EDX, and Mössbauer spectroscopy data, we estimated the inversion degree parameter (γ), which should be interpreted as reflecting general trends rather than absolute values. Particular attention was given to estimating γ for the undoped CFO sample, as well as the Mn-3 and Cu-3 samples. The inversion degree for the undoped sample (γ_CFO_) was 0.75. In comparison, the Cu-3 sample exhibited a slightly lower value of about 0.70, while the Mn-3 sample showed a value close to 1.0, opposite to the trend observed in the Cu-doped sample. These findings are consistent with the Fe L_2_-edge XAS data.

### 3.5 The Analysis of Textural Properties Using N_2_ Physisorption Isotherms

The textural properties of the samples were evaluated using N_2_ physisorption isotherms and BJH pore size distribution curves (Figures S8 and 5a,b and Tables S2 and S3). The samples demonstrate the type IV isotherms with an H3 hysteresis loop. It can be seen that the BET surface area differs between the samples annealed at 300 and 400 °C. Typically, the surface areas of mesoporous metal oxides range from 90 to 150 m^2^/g.^49^ In our study, the BET surface area of the samples annealed at 300 °C falls within a similar range of 98.9–159.8 m^2^/g, whereas a further increase in annealing temperature to 400 °C results in a decrease in BET surface area to 49.5–115.1 m^2^/g. However, the samples exhibit BET surface area similar to or even higher than those obtained with a similar solvothermal method.^9,50^ The annealing at 400 °C decreases the BET surface area and volume of mesopores of all samples by approximately 30% and increases the average pore diameter by approximately 25% (Table S3). The increase in pore diameter (hence a decrease in the number of pores) results in decreased pore volume. All samples demonstrate a wide pore size distribution with pore sizes ranging from 1.5 to 20 nm (Figure 5b). The increase in the specific surface area (Figure 5c) and pore volume can be attributed to the open pores formed by the nanoparticles within the microspheres,^51^ as evidenced by the TEM results (Figure 5d). The PSD analysis of the Mn-3 sample reveals two distinct peaks at 5 and 9.5 nm. In contrast, the PSD curve for the Mn-1 sample indicates a single peak centered at 8 nm. In the case of undoped cobalt ferrite, a less pronounced peak is observed at 3.5 nm, with a stronger secondary peak at 12 nm. Cu-containing samples annealed at 400 °C show a similar trend, with the difference that the maximum on the PSD curve shifts toward larger mesopores for all samples, and their volume is smaller compared to the samples annealed at 300 °C. A feature of the Cu-3 sample is that mesopores of 2–3.5 nm in size also appeared, which were not observed in the Cu-1 sample. On the other hand, the maximum on the PSD curve shifted to 12.4 nm for all samples (Figure 5b). These changes caused a decrease in the specific surface area of the samples (Table S3), since it is a well-known fact that the larger the pores and the larger the particles, the smaller the specific surface area. Thus, the proposed synthesis method confirms its suitability for obtaining mesoporous samples with a highly developed surface.

**Figure 5:**
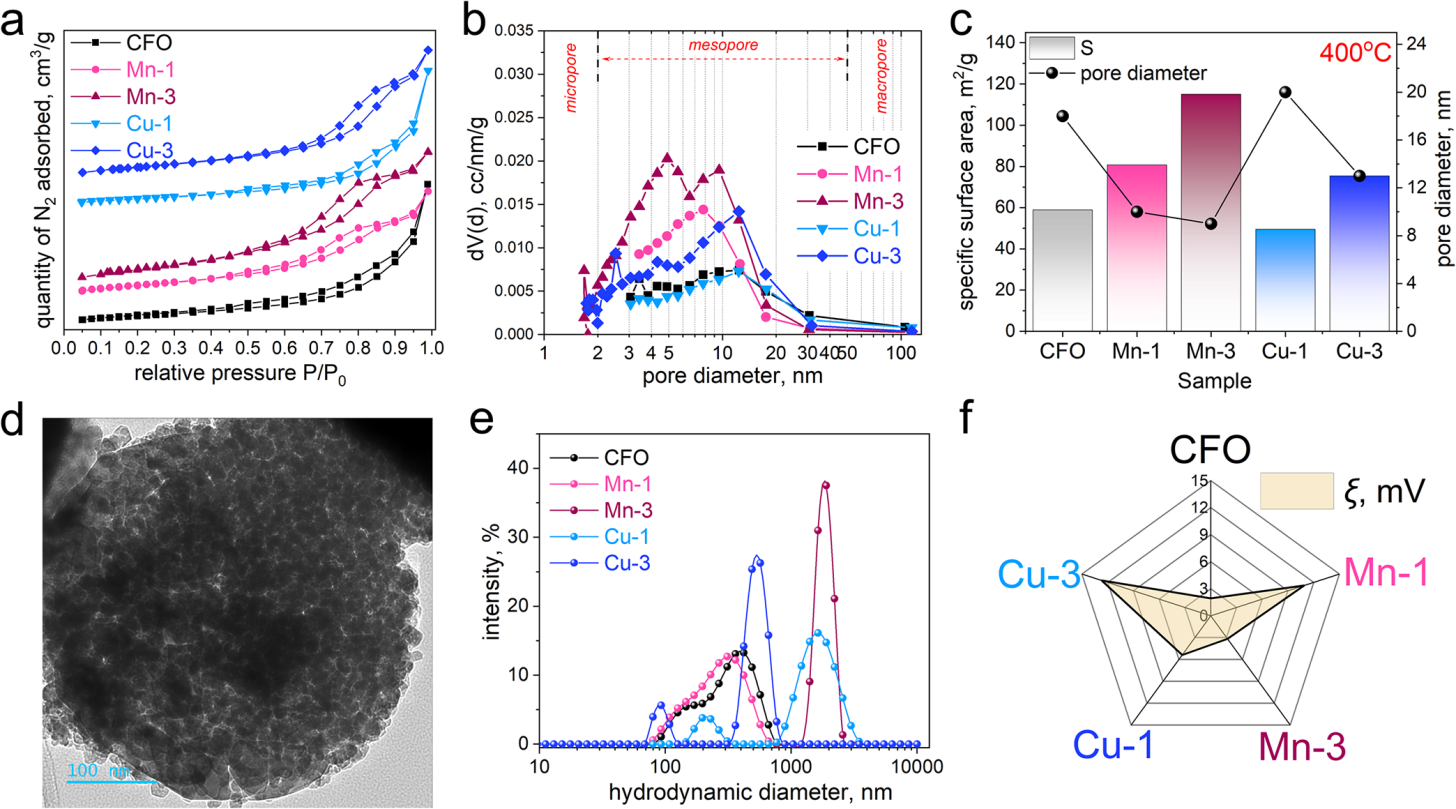
(a) N_2_ adsorption/desorption isotherms and (b) pore size distributions for ferrite samples annealed at 400 °C. (c) SSA and pore size. (d) TEM image of the Mn-3 sample. (e) hydrodynamic diameter distributions and (f) ξ-potentials.

### 3.6 Particle Size and Stability in Aqueous Dispersions

Dynamic light scattering (DLS) analysis demonstrated that introducing Mn and Cu into the spinel ferrite crystal lattice affects particle hydrodynamic size and surface charge (Figure 5e and Table S4). The Cu-3 sample demonstrates enhanced stability due to a higher ξ-potential (Figures 5f and S9a). Polydispersity index (PDI) shows that doping influences both particle size and their distribution (Table S4). All samples, except Mn-3, exhibit high PDI, indicating a broad particle-size distribution and reduced uniformity. The Cu-3 sample, even though it has the highest zeta potential, exhibits the widest particle size distribution (PDI = 0.5546), possibly due to the presence of small and aggregated particles (Table S4). The Mn-3 sample exhibits a low PDI (0.1552), indicating the presence of uniformly distributed large aggregates, likely due to its low surface charge (ξ = 3.168 mV). DLS analysis of the Cu-1 and Cu-3 samples revealed bimodal size distributions. For the Cu-1 sample, a primary peak at 1710 nm (87.6% intensity) and a minor secondary peak at 216.1 nm (12.4% intensity) are observed. These results correlate well with the particles’ morphology, as confirmed by SEM (Figure S9b–d).

The measured ξ-potential values offer insight into the colloidal stability of NPs and their interaction with each other in liquids. Low values of ξ-potentials indicate NP aggregation, most likely due to their magnetic properties. The undoped CFO sample exhibits a very low ξ-potential of 1.943 mV, leading to rapid settling in aqueous solutions. It means that the pH of the solution containing the CFO sample is very close to its isoelectric point, which usually is in the range of 6.5–7.6 for most spinel ferrites.^52,53^ Mn and Cu doping leads to an increase in the ξ-potential (10.9 mV for Mn-1 and 12.73 mV for Cu-3), indicating that the doping improves the surface charge and those samples are better electrostatically stabilized in aqueous solutions (see Supporting Information, Video S1). ξ-potential increased in the series: CFO < Cu-1 < Mn-3 < Mn-1 < Cu-3. The substitution with Mn and Cu alters the surface potential, enhancing the stability of NPs dispersions compared to undoped cobalt ferrite. Such effects are potentially beneficial for adsorption or catalytic applications.

### 3.7 Adsorption Properties and Fenton-like Activity

Two model pollutants, the oxytetracycline antibiotic (OTC) and Congo red dye (CR), were used to explore the adsorption and catalytic bifunctionality of synthesized spinels, annealed at 400 °C. The study of adsorption and catalytic properties involved two different approaches. First, the model pollutant was directly oxidized with H_2_O_2_ in the presence of the spinel catalyst, without a prior adsorption step. The second approach utilized a two-step process that included an initial preadsorption of the pollutant onto the catalyst surface before the oxidation step.

The oxytetracycline degradation curves are presented in Figure 6a. It should be noted that H_2_O_2_ alone does not degrade OTC in 2 h (Figure S10a). The most active sample, Mn-3, can remove 98% of OTC within 30 min (Figure 6b). The Mn-1 and Cu-3 samples remove 87% and 84% of OTC, respectively. At the same time, cobalt ferrite can remove only 62% of OTC. After 90 min, the activity of the samples can be lined up as follows: Mn-3 (99%) > Mn-1 ∼ Cu-3 (94%) > Cu-1 (85%) > CFO (69%) (Figure 6b). The Mn- and Cu-doping increases the catalytic activity of the cobalt ferrite by 23–37%. Because the kinetic data do not fit well with the pseudo-first- and pseudo-second-order models, the initial reaction rate, *r*_0_, was used as the primary quantitative parameter for comparing the samples. Figure 6c shows that the Mn-3 sample exhibits the highest *r*_0_ value, 0.233 ± 0.03 mg/(L·min), 1.73 times higher than that of the CFO reference. The measured activity follows the series: Mn-3 > Mn-1 > Cu-3 > Cu-1 > CFO, demonstrating enhanced activity of Mn- and Cu-doped ferrites compared to undoped ones.

**Figure 6:**
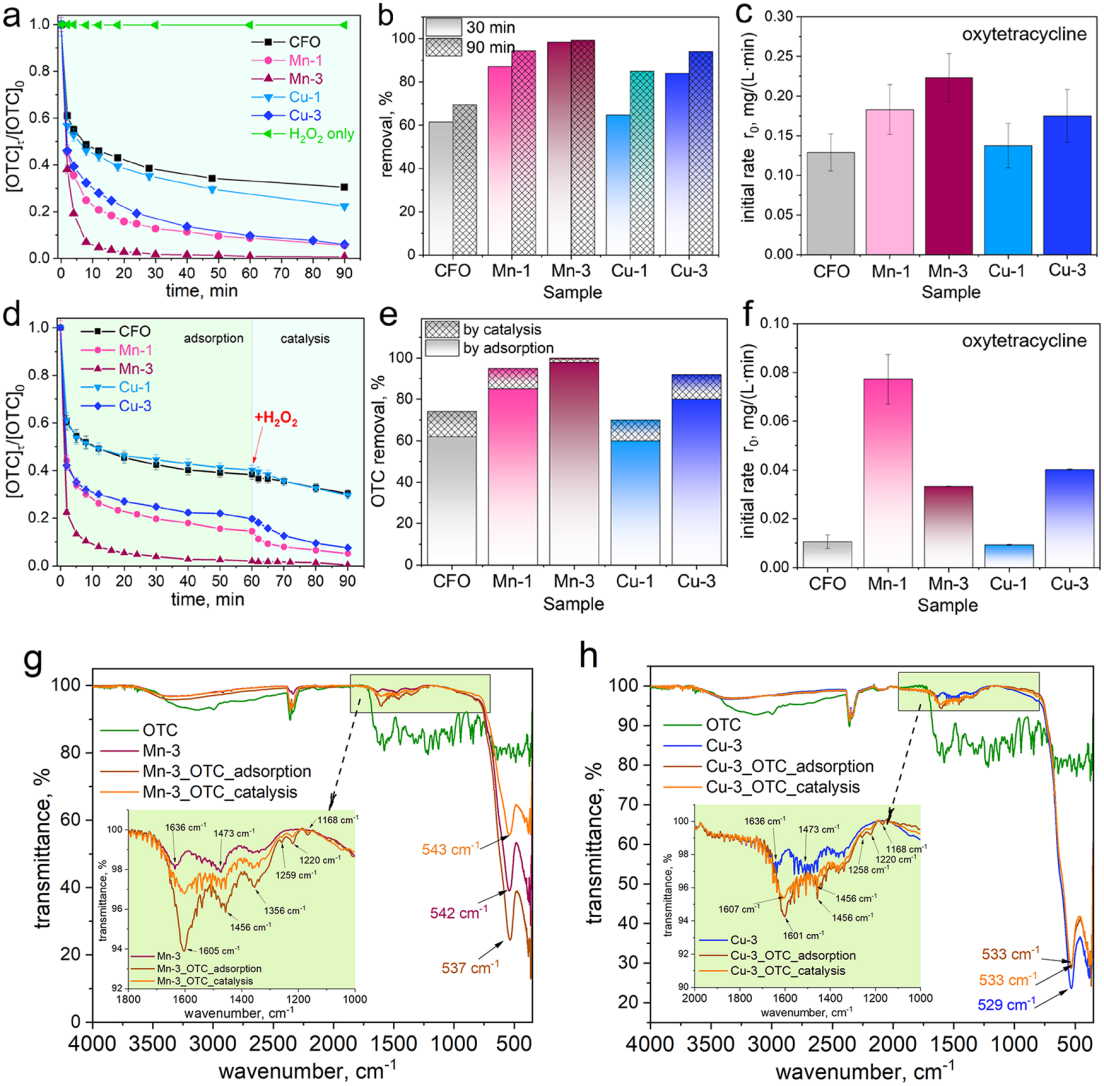
(a) Kinetic curves demonstrating the catalytic degradation of OTC using Mn- and Cu-containing cobalt ferrites and H_2_O_2_; (b) removal of OTC by catalysis only; (c) the initial rate of OTC degradation; (d) kinetic curves demonstrating the adsorption of OTC (during 60 min) and degradation of OTC (during 30 min) using Mn- and Cu-containing cobalt ferrites and H_2_O_2_; (e) removal of OTC by adsorption and catalysis (in %); (f) the initial rate of OTC degradation after adsorption; (g, h) FTIR spectra of (g) Mn-3 and (h) Cu-3 samples before adsorption, after adsorption or after catalytic degradation of OTC (conditions: [OTC] = 20 mg/L, *V* = 25 mL, *m* = 25 mg, [H_2_O_2_] = 10 mM, pH ∼ 7, *T* = 20 °C).

The experiments, which included separate adsorption and catalysis steps, were performed to study the adsorption properties of the samples and their impact on overall activity (Figure 6d). The process involved 60 min of adsorption (until saturation), followed by 30 min catalytic reaction initiated by the addition of H_2_O_2_. In this case, the overall OTC removal is very similar in both cases (Figure 6b,6e). The results evidence that adsorption plays a significant role in the OTC removal. The Mn-3 sample captures OTC molecules more effectively on its surface. The process is surface-driven in this case: H_2_O_2_ reacts primarily with adsorbed OTC, rather than with the OTC in bulk solution (*vide infra*). Figure 6e depicts that adsorption is the dominant removal mechanism, accounting for 62% (for CFO) to 98% (for Mn-3) of the total OTC removal. Catalysis by peroxidation involves 2% (for CFO) to 12% (for Mn-1) of OTC removal. Mn-3 shows 100% of total OTC removal – most through adsorption, and a small fraction (2%) through catalysis. The initial degradation rate of OTC during the catalytic stage (Figure 6f) shows that Mn-1 and Cu-3 samples exhibits the highest efficiency. The difference in the initial rates between the samples is caused by different active sites on the surface, arising from Mn or Cu. The highest initial rate indicates more reactive surface centers, and also better activation of H_2_O_2_. Finally, it can be concluded that higher Mn or Cu contents improve both the adsorption and catalytic functionalities of cobalt ferrite. However, Cu-based systems remain less active than Mn-based systems. It can also be concluded that preadsorption does results in any significant loss of activity. If the catalyst is deactivated or saturated during adsorption, a less efficient degradation would be expected in the second case. Thus, the adsorbed OTC is still reactive and can undergo catalytic degradation. Adsorption is a rather dominating step, not the oxidation reaction. Moreover, H_2_O_2_ must be present at the right moment, but not necessarily from the beginning of the process. FTIR spectra of the most active samples, Mn-3 and Cu-3, before and after OTC adsorption/catalytic degradation are presented in Figure 6g,h and the detailed explanation is provided in the Supporting Information (Text S6). Concluding, the catalytic degradation of OTC was effective, and the catalyst surface remained relatively clean, with no significant fouling from residual byproducts.

The Congo Red adsorption/degradation experiments are presented in Figure 7. The results demonstrate that the most effective catalysts are Mn-3 and Cu-3 (Figure 7a). Specifically, the Mn-3 sample achieves complete degradation of CR in 40 min, while the Cu-3 sample fully decomposes CR in 60 min (Figure 7a). The removal efficiency is notably rapid during the first 15 min and then gradually slows down. A fast catalytic oxidation of CR occurs primarily within the initial 30 min, followed by a slower removal (Figure 7b). It is important to note that all doped samples show enhanced activity compared to undoped cobalt ferrite. For the Mn-3 catalyst, the initial degradation rate was *r*_0_ = 0.1248 mg/(L·min), whereas for the Cu-3 catalyst, it was *r*_0_ = 0.1104 mg/(L·min) (Figure 7c).

**Figure 7:**
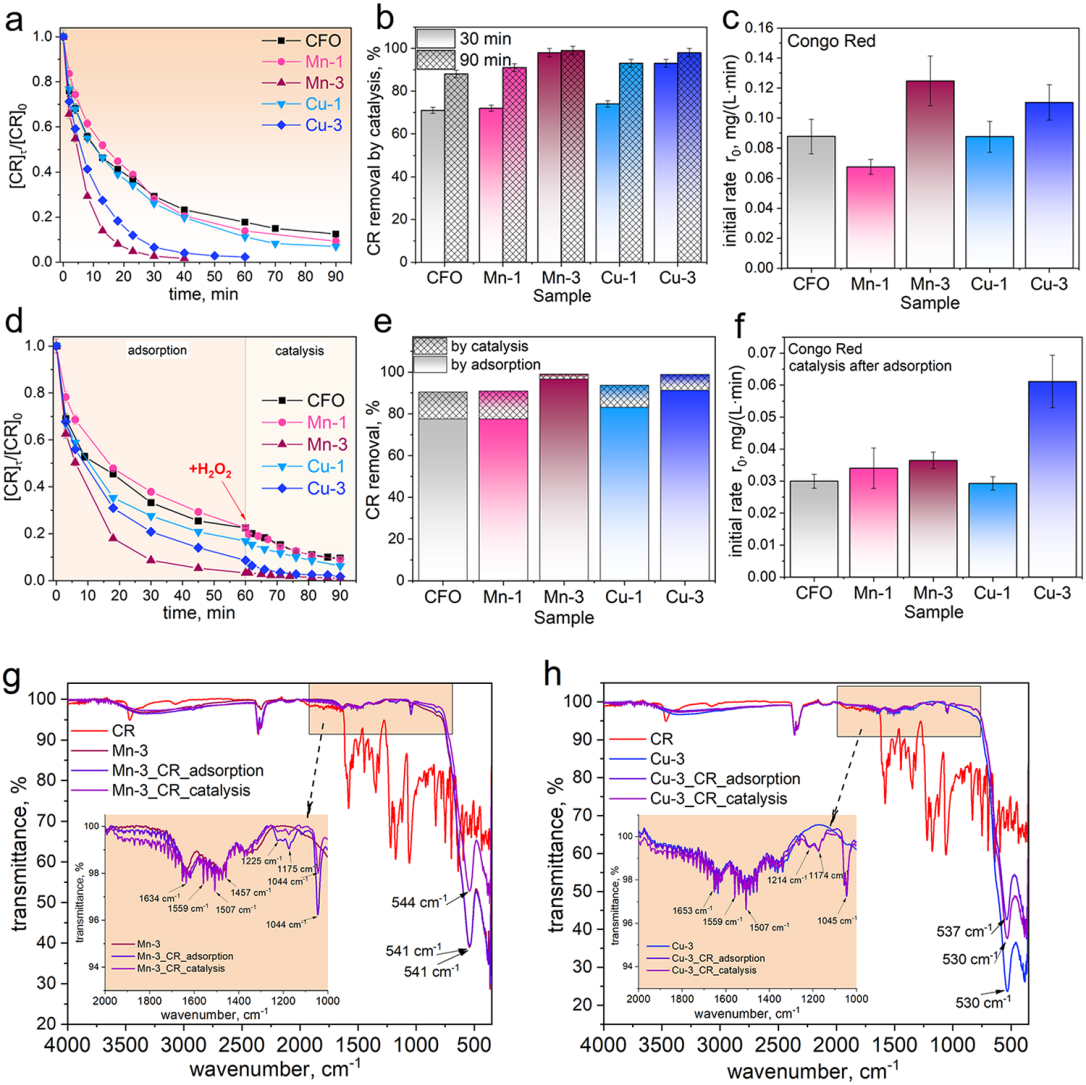
(a) Kinetic curves demonstrating the catalytic degradation of CR using Mn- and Cu-containing cobalt ferrites and H_2_O_2_; (b) removal of CR by catalysis only; (c) the initial rate of CR degradation; (d) kinetic curves demonstrating the adsorption of CR (during 60 min) and degradation of CR (during 30 min) using Mn- and Cu-containing cobalt ferrites and H_2_O_2_; (e) removal of CR by adsorption and catalysis (in %); (f) the initial rate of CR degradation after adsorption. FTIR spectra of (g) Mn-3 and (h) Cu-3 samples before adsorption, after adsorption, or after catalytic degradation of CR (conditions: [CR] = 20 mg/L, *V* = 25 mL, *m* = 25 mg, [H_2_O_2_] = 10 mM, pH ∼ 7, *T* = 20 °C).

The second approach used sequential adsorption (for 60 min) and catalytic degradation in the presence of H_2_O_2_ (for 30 min) (Figure 7d). Again, the Mn-3 sample shows the highest removal activity. All Mn- and Cu-containing samples exhibited high CR removal efficiency, primarily driven by a strong adsorption affinity (Figure 7e). Still, the extent of catalytic contribution varied significantly depending on the dopant type and content. Among the tested materials, Mn-3 achieved nearly complete CR removal (99.1%), attributed almost entirely to adsorption, with a negligible catalytic enhancement. Mn-1 displayed similarly high adsorption-driven removal, with a catalytic contribution of 13.4%. This suggests that Mn-doping enhances the surface affinity of CR molecules, likely due to improved textural properties or more uniform surface charge distribution. Among the Cu-doped ferrites, Cu-3 exhibited the highest catalytic degradation rate (*r*_0_ ≈ 0.0611 ± 0.0082 mg/(L·min)) (Figure 7f), significantly surpassing the activity of the other samples. This improvement is attributed to enhanced redox cycling (Cu^2+^/Cu^+^) and more efficient generation of HO^•^ radicals in the presence of H_2_O_2_. The higher Cu content in Cu-3 appears optimal for facilitating electron transfer and sustaining the catalytic cycle. Cu-1, with lower Cu loading, showed inferior performance, indicating insufficient availability of active sites. While Mn-doped materials were more effective for CR adsorption, Cu-3 emerged as the most promising catalyst for advanced oxidation, offering a synergistic balance between dye uptake and catalytic degradation. These findings highlight the critical role of dopant selection and loading in tuning the surface and redox properties of spinel ferrite catalysts for wastewater treatment applications. The adsorption of Congo Red onto the Cu- and Mn-containing ferrites was further confirmed by FTIR spectroscopy (Figure 7g,h). The detailed explanation is provided in the Supporting Information (Text S7). It can be concluded that the catalyst retains structural stability during the Fenton-like conditions, and the core M–O bonds are not chemically altered by the presence of residual organics. The hollow structure and surface chemistry (due to Mn or Cu doping) greatly enhance CR and OTC adsorption, likely due to the increased surface area, surface charge interactions, and coordination between the pollutant’s functional groups (like amines or ketones) and metal ions (Mn^2+^, Cu^2+^).

To separate surface-area-controlled adsorption from intrinsic catalytic activity, we normalized the experimentally determined initial rates of pollutant removal to the BET-specific surface area (Figure S11a,b). The Cu-doped samples still display higher catalytic efficiency per unit area, indicating that Cu substitution enhances H_2_O_2_ activation and radical-mediated degradation in addition to adsorption. Mn-substitution increases surface area and therefore increases the amount of CR or OTC removed by adsorption, while contributing little to per-area H_2_O_2_ activation. The results demonstrate that the materials are truly bifunctional: adsorption controls the macroscopic removal capacity and kinetics, whereas dopant-dependent redox chemistry provides a secondary, mechanistically important catalytic route that degrades pollutants via radical pathways. The data is consistent with Cu-mediated redox cycling observed in XAS and XPS and with the dominant HO^•^ signal in EPR studies (see Section 3.8).

### 3.8 Mechanistic Insights into Adsorption and Fenton-like Catalysis

XAS, XPS, Raman, and EPR analyses were collectively used to better understand the Fenton-like catalytic mechanism. Each technique provided insight into a different aspect of the process: Raman revealed the structure and possible defects; XPS and XAS tracked changes in metal oxidation states and vacancies; while EPR identified the reactive species involved.

XAS analysis of bulk metal redox states is presented in Figure 8a–f. After the catalytic process, a change in the Co^2+^/Co^3+^ ratio is observed (Figure 8a,d). Both the Mn-3 and Cu-3 samples exhibit the same trend: a decrease in the Co^3+^ population accompanied by an increase in the Co^2+^ population. Although it is difficult to determine whether the observed Co^2+^ originates from Co_3_O_4_ or cobalt ferrite, Co^3+^ is more commonly associated with Co_3_O_4_. Therefore, it is reasonable to assume that the reduction of cobalt predominantly occurs in the Co_3_O_4_ phase rather than in the cobalt ferrite phase. Figure 8b shows the evolution of the Mn L_3_-edge spectra after catalysis, compared to the spectrum recorded before catalysis. Following the catalysis, a decrease in the Mn^2+^ population is observed, accompanied by a corresponding increase in the Mn^3+^ population. XAS measurements at the Cu L_3_-edge reveal a predominant signal corresponding to Cu^2+^, with a minor contribution from Cu^+^ (Figure 8e). After the catalytic reaction, the amount of Cu^+^ appears to increase, particularly following OTC degradation. However, its contribution remains minor compared to the clearly dominant signal from Cu^2+^ ions. The catalytic process does not appear to significantly affect the oxidation state of Fe, as no notable changes are observed in the XAS spectra (Figure 8c,f). The most significant spectral changes at the Co edge indicate that cobalt actively participates as a redox center in the catalytic mechanism. In contrast, the Fe edge shows no notable changes, suggesting that iron remains chemically stable and does not engage in redox cycling. Alterations observed at the Mn edge further support its involvement as a redox-active site. Although a slight increase in Cu^+^ is detected after catalysis, its overall low concentration limits its potential contribution to the catalytic activity. These findings confirm that Co, Mn, and Cu function as redox-active elements, while Fe remains redox-inert. This could be explained by the fact that, in the spinel structure, Fe^3+^ predominantly occupies octahedral sites and remains in a thermodynamically stable oxidation state under the reaction conditions studied. The surrounding lattice oxygen, together with Co, Mn, and Cu ions, stabilizes the electronic configuration of Fe^3+^ ions through strong Fe–O covalent interactions.^54^ This structural stabilization suppresses Fe^3+^/Fe^2+^ redox cycling typical of conventional Fenton systems. Instead, Co, Mn and Cu ions act as the primary redox-active centers, facilitating electron transfer and ROS generation. Therefore, Fe plays the role of a redox-inert structural stabilizer rather than that of an active participant in ROS production.

**Figure 8:**
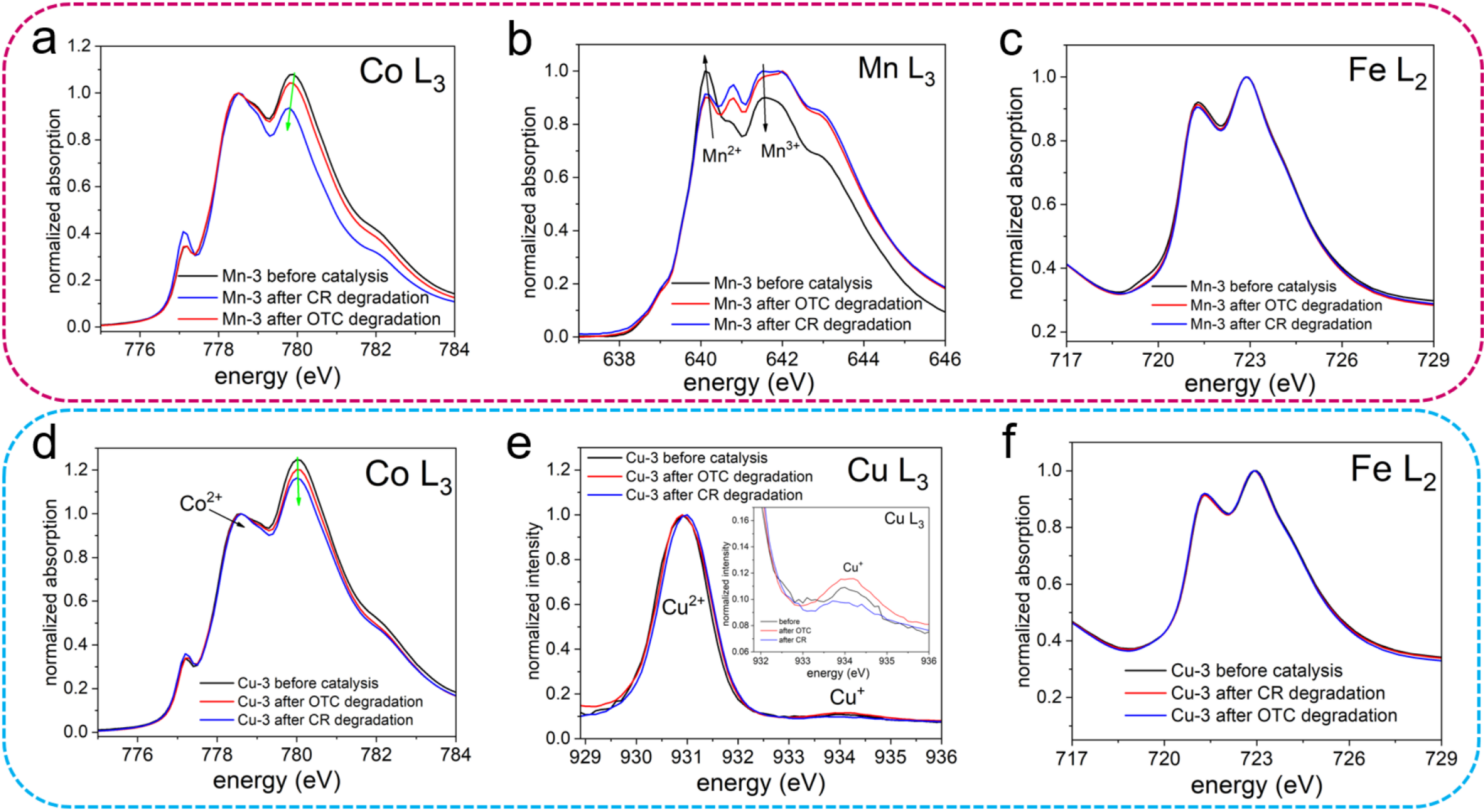
XAS at metal L-edges of (a–c) Mn-3 and (d-f) Cu-3 samples before and after catalysis: (a, d) Co L_3_-edge of the (a) Mn-3 and (d) Cu-3 samples. (b) Mn L_3_-edge of the Mn-3 sample. (e) Cu L_3_-edge of the Cu-3 sample. (c,f) Fe L_2_-edge of (c) Mn-3 and (f) Cu-3 samples.

Figure S12 shows wide-range survey scans collected for the model pollutants and selected ferrites before/after adsorption or catalysis. Acquired scans for OTC/CR are dominated by carbon and oxygen elements present in their structure, with some signals from nitrogen, and a sulfur line for CR, without additional impurities. Figures S13 and S14 show XPS analysis of surface redox behavior and oxygen species for the Mn-3 and Cu-3 samples, which demonstrated the highest adsorption/catalytic degradation activity.

The spectra of the ferrites are exhibit strong signals from Co, Fe, O, and either Cu or Mn, along with weak signals from carbon contaminants, likely due to air exposure. No traces of sulfur or nitrogen were detected. Survey scans of the ferrite samples after CR and OTC adsorption/catalytic degradation show subtle changes in the intensity of these lines along with slight enhancements in the N 1s and S 2p regions – features characteristic of the adsorbed pollutants. To reliably determine the chemical states and enable quantification, high-resolution XPS spectra were collected for all detected elements in the Co 2p_3/2_, Fe 2p_3/2_, Mn 2p_3/2_, Cu 2p_3/2_, O 1s, C 1s, N 1s, and S 2p regions, as shown in Figures S13 and S14.

The Co 2p_3/2_ region for the catalysts was fitted with five components (Figure S13a). The first peak, located at 779.6 eV, corresponds to a mixed presence of Co^2+^ and Co^3+^ cations, similar to those found in Co_3_O_4_ species. The remaining four Co 2p_3/2_ lines, observed in the 782–789 eV range, are attributed to multiplet splitting phenomena typical of Co^2+^ ions.^55^ The Fe 2p_3/2_ spectra (Figure S13b) are similar across all ferrite samples and were fitted with six components. The first peak at 709.4 eV corresponds to the Fe^3+^ oxidation state. Four peaks between 710 and 715 eV arise from multiplet splitting, while the shifted shakeup satellite at 717.5 eV provides further evidence for the presence of Fe^3+^ cations.^56^

The Mn 2p_3/2_ spectra of Mn-doped ferrites (Figure S13c) were fitted with up to five components. The main peak, centered at 640.3 eV, corresponds to the Mn^2+^ oxidation state. The four additional peaks between 641 and 646 eV arise from multiplet splitting phenomena; plausibly, and the presence of Mn^3+^ species at about 641.2 eV is also possible.^57^ The Cu 2p_3/2_ spectra collected for Cu-containing samples were fitted with up to six components (Figure S13d). The primary peak at 932.9 eV indicates the presence of Cu^+^ oxidation state, whereas the five peaks starting from 934.7 eV represent Cu^2+^ ions.^58^

The O 1s spectra (Figure S14a) are similar for all catalysts and were fitted using three lines, with the first line centered at 530.0 eV, which indicates the presence of M–O bonds (O–Co, O–Fe, O–Mn, O–Cu), the second line at 531.9 eV represents defective oxygen in metal oxides and/or O=C and/or O–S type bonds, and the last line found at 533.1 eV can originate either from O–H and/or O–C type bonds and/or adsorbed H_2_O.^59^ In ferrite samples, changes in XPS spectra following adsorption and/or catalysis processes can be observed, including a decrease in signals from detected metals and the first line on oxygen representing oxides, an increase in signals in the C 1s region, particularly within the C=C/C–C and C–C/C–N bonds (Figure S14b), and the appearance of lines from π–π shakeup satellites, as well as new signals from nitrogen (Figure S14c) and sulfur (Figure S14d). The intensity of these changes is greater for Mn-doped samples and more pronounced after the catalysis step. The detected N 1s regions were fitted with two lines (Figure S14c), i.e., first at 400.2 eV originating from amine-type groups and the second at 402.3 eV comes from protonated amine and/or N­(CH_3_)_3_^+^ species.^60^ In turn, the S 2p spectra of samples after adsorption/catalytic degradation of CR dye (Figure S14d) were fitted with one doublet structure (p_3/2_-p_1/2_ separation equals 1.16 eV) with the main 2p_3/2_ line centered at 168.5 eV, which represents SO_3_^2–^ ions.^59^

Thus, all the described changes confirm the presence of CR and/or OTC molecules on the ferrite surface. It should be noticed that this surface adsorption leads to a noticeable attenuation of the ferrite-related elemental signals, especially in the Mn-containing samples after catalysis, suggesting a potentially higher efficiency of pollutant removal in these systems. Additionally, subtle shifts in binding energy observed after adsorption and catalysis are within the spectrometer’s resolution. Therefore, XPS studies do not provide direct evidence of the formation of new states or chemical bonds. Instead, the observed interactions are more likely attributed to physical phenomena such as hydrogen bonding, electrostatic forces, or π–π interactions.

Figure 9a presents Raman spectra for CFO and the two most active samples, Mn-3 and Cu-3. Typically, the five Raman-active modes in ferrites’ spectra, arising from the cubic Fd3m space group symmetry, appear at ∼190–220 cm^–1^ (T_2g_(1) mode, corresponding to M–O lattice vibrations), ∼280–320 cm^–1^ (E_g_ mode, corresponding to symmetric bending of O–Fe–O bonds), around 380–470 cm^–1^ and 490–590 cm^–1^ (T_2g_(2) and T_2g_(3) modes related to asymmetric bending vibrations in octahedral sites), and around 650–690 cm^–1^ (A_1g_ mode, attributed to symmetric stretching of M–O bonds in tetrahedral sites).^61–63^ For all three samples, two bands are visible at ∼470 cm^–1^ and ∼670 cm^–1^, which correspond to T_2g_(2) mode, demonstrating the vibration of the spinel structure, and A_1g_ mode, demonstrating the stretching vibrations of the Fe–O and M–O bonds in tetrahedral sites, respectively. For CFO and Cu-3, a band at ∼190 cm^–1^ appears, corresponding to T_2g_(1) mode.^64^ For the Mn-3 sample, the bands at 292 cm^–1^ and 543 cm^–1^ are visible, corresponding to E_g_ and T_2g_(3) modes, respectively.

**Figure 9:**
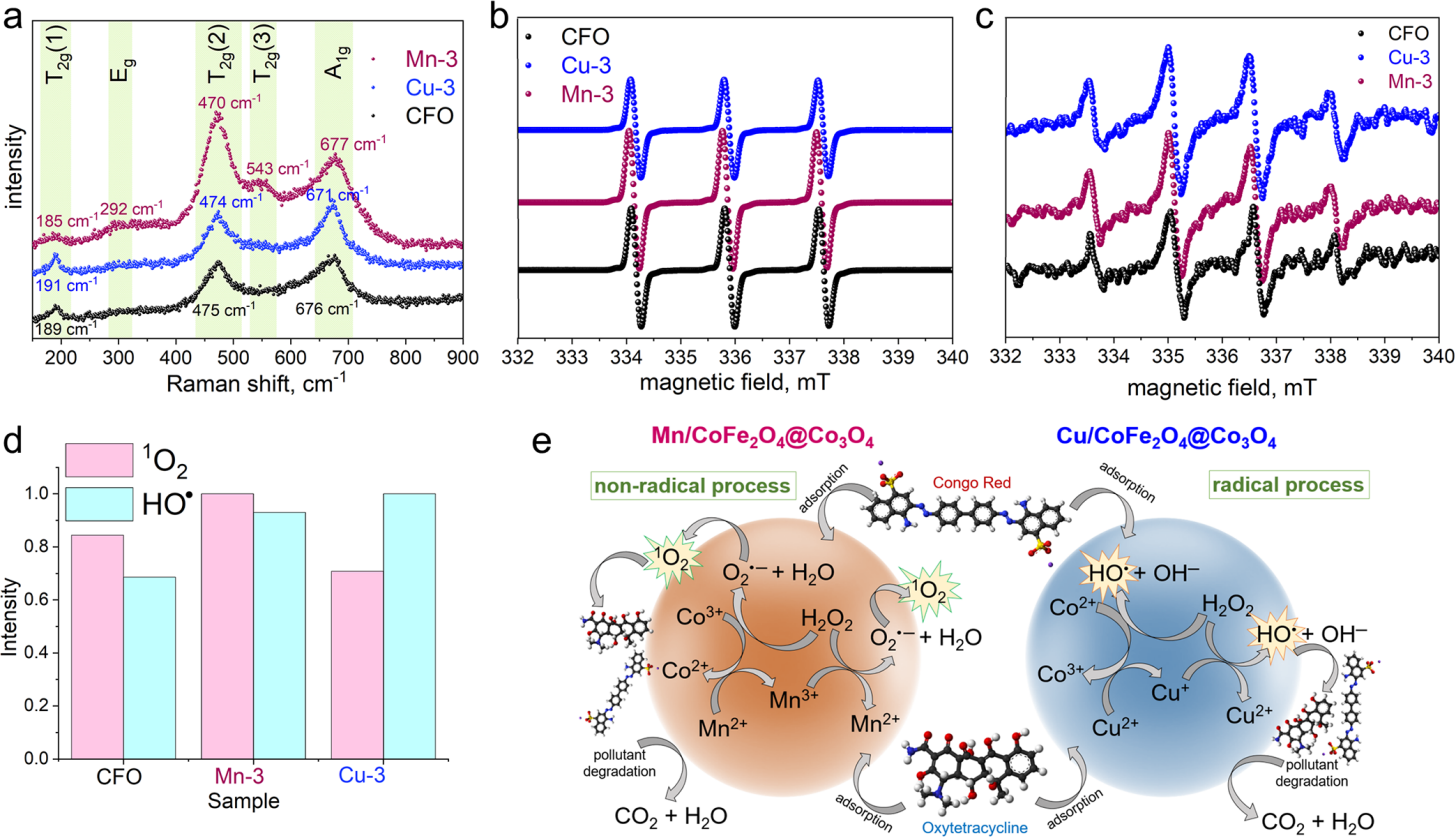
(a) Raman spectra of the CFO, Mn-3, and Cu-3 samples. (b,c) EPR spectra recorded at room temperature after the reaction of the tested samples with H_2_O_2_ in the presence of (b) TEMP and (c) DMPO spin traps. (d) The comparative intensity of signals showing the HO^•^ and ^1^O_2_ formation. (e) The plausible mechanism of Fenton-like degradation of OTC and CR using Mn- and Cu-containing CoFe_2_O_4_@Co_3_O_4_.

It should be noted that doping with Mn or Cu increases Raman intensity, particularly for the T_2g_(2) and A_1g_ modes. This suggests increased polarizability and possible lattice distortion resulting from substitutional doping, which in turn improves both adsorption capacity (by modifying surface sites and charge distribution) and Fenton-like catalytic activity (by promoting redox-active metal centers and electron transfer). The modified M–O vibrational environment supports enhanced H_2_O_2_ activation efficiency, which aligns with the higher degradation rates observed in the catalytic experiments (see Section 3.7). Additionally, weak but distinct features in the range of ∼480–525 cm^–1^ and ∼620 cm^–1^, particularly evident in Mn-3 and Cu-3, suggest the presence of a secondary Co_3_O_4_ phase, as these bands correspond to its characteristic E_g_ and F_2g_(3) modes. This observation supports conclusions drawn from XRD and XAS analyses, which also indicate the coexistence of Co_3_O_4_ alongside the primary phase. The increased broadness and slight asymmetry of the A_1g_ mode near 670 cm^–1^, particularly in the Mn- and Cu-containing samples, may also result from overlapping contributions of Co_3_O_4_, further supporting the presence of structural inhomogeneity. Additionally, the broad background signal and mode broadening observed across the entire Raman spectrum – especially in Mn-3, suggest the formation of oxygen vacancies, which are known to play an important role in ROS formation during Fenton-like reactions.

EPR and spin trapping techniques were used to confirm a Fenton-like catalytic mechanism. An additional observation was made from UV–vis spectra. It was noted that when the Mn-3 sample was used as a Fenton-like catalyst, there was no residual H_2_O_2_ present in the system following the degradation of the Congo Red or oxytetracycline. In contrast, when the Cu-3 sample served as the Fenton-like catalyst, residual H_2_O_2_ remained in the system after pollutant degradation. Thus, the reasons for this difference were investigated through EPR studies (Figure 9b,c). In all examined samples, the generation of both HO^•^ radicals and singlet oxygen ^1^O_2_ was detected. This was evidenced by the appearance of four spectral lines with an intensity ratio of 1:2:2:1 for samples containing DMPO (Figure 9c) and three lines of the same intensity for the reactions involving TEMP (Figure 9b). The Spin Hamiltonian parameters obtained from computer simulations for the DMPO–OH adducts revealed *g*_iso_ = 2.0052, *a*_iso_^N^ = *a*_iso_^H^ = 1.5 mT, while the parameters for the reaction with TEMP indicated *g*_iso_ = 2.0047 and *a*_iso_^N^ = 1.73 mT. Surprisingly, the highest activity for hydroxyl radical generation is observed in the Cu-3 sample; however, this sample displays the lowest activity in the formation of singlet oxygen (Figure 9c). In contrast, the Mn-3 sample exhibits superior activity in the ^1^O_2_ generation.

The plausible mechanism of Fenton-like degradation of OTC and CR using Mn- and Cu-containing CoFe_2_O_4_@Co_3_O_4_ is presented in Figure 9e. It is noteworthy that CR and OTC removal is surface-driven: strong adsorption on the hollow, high-surface-area spheres concentrates CR or OTC at the samples’ surface, bringing substrates into tight contact with metal sites and oxygen vacancies. In turn, oxygen vacancies enhance H_2_O_2_ adsorption/activation and facilitate electron transfer between metal centers and peroxide/pollutant. Moreover, in the studied Mn-CoFe_2_O_4_@Co_3_O_4_ and Cu-CoFe_2_O_4_@Co_3_O_4_ systems, the Lewis acidity of the metal ions is actually central to how the Fenton-like catalysis proceeds.^65^ They can coordinate electron-rich species, such as H_2_O_2_, polarize O–O bond and make it easier to break, generating ROS. The main steps include: (i) H_2_O_2_ adsorption/activation; (ii) ROS generation; (iii) CR/OTC adsorption/oxidation; (iv) redox cycling. It is expected that H_2_O_2_ is adsorbed on the surface redox centers (oxygen vacancies) and undergo activation by electron transfer at Co/Mn/Cu centers. The conversion of H_2_O_2_ into ROS (what is evidenced by EPR trapping) is possible through the two ways depending on the metal active center and its redox state: (i) via one-electron reduction pathway into hydroxyl radicals HO^•^ at M^2+^ active sites; or (ii) via two-electron or sequential pathways and interconversion reactions into superoxide/hydroperoxyl (O_2_^•–^/HOO^•^) and subsequently singlet oxygen (^1^O_2_). Then, the adsorbed CR/OTC molecules are attacked by the ROS formed, leading to their degradation. Finally, partial regeneration of metal centers occurs via subsequent reactions with H_2_O_2_, dissolved O_2_, or electron transfer from adsorbed organics, allowing catalytic turnover. XAS after catalysis shows changes in metal oxidation states at Co, Mn and Cu sites. In particular, XAS shows a decrease in Co^3+^ and an increase in Co^2+^ after catalysis. This indicates a reduction of Co at the surface during reaction (i.e., Co^3+^ → Co^2+^), consistent with Co sites accepting electrons or being reduced by H_2_O_2_ (or by electrons from the adsorbed organic molecules) as they participate in ROS generation. Because Co^3+^ is abundant in Co_3_O_4_, the data suggest the Co_3_O_4_ shell is the main location of cobalt redox activity.^66^

The observed XAS shifts connected with Mn^2+^ → Mn^3+^ and Co^3+^ → Co^2+^ changes in Mn-3 sample after catalytic reaction, together with stronger ^1^O_2_ formation (compared to Cu-3 sample), indicate that Mn centers favor pathways that produce O_2_^•–^/HOO^•^ and promote transformation of these species into ^1^O_2_ (either directly or via surface-mediated disproportionation):^67^

$${\mathrm{Mn}}^{2+}\to {\mathrm{Mn}}^{3+}+e^{-}$$

$$O_{2}+e^{-}\to {O_{2}}^{•-}$$

$${O_{2}}^{•-}+H^{+}\to {\mathrm{HOO}}^{•}$$

HOO•+O2•−→O21+OH−+O2

This favors ^1^O_2_-dominated oxidation pathways, which are often more selective than to HO^•^ radical pathways.

The increase in Cu^+^ (noted from XAS data, especially after OTC tests) indicates reduction of Cu^2+^ to Cu^+^ during catalysis. Cu^+^ is a known efficient activator of H_2_O_2_ (Fenton-like), which explains the strong HO^•^ signal seen for the Cu-3 sample by EPR. Probably, Cu^2+^ (even at low concentration) undergoes reduction to Cu^+^ and participates in the classical Fenton-like process:

$${\mathrm{Cu}}^{+}+H_{2}O_{2}\to {\mathrm{Cu}}^{2+}+{\mathrm{HO}}^{•}+{\mathrm{OH}}^{-}$$

At the same time, the Co sites (from Co_3_O_4_ shell) also contribute via Co^2+^/Co^3+^ cycling, producing additional HO^•^:^66^

$${\mathrm{Co}}^{3+}+e^{-}\to {\mathrm{Co}}^{2+}$$

$${\mathrm{Co}}^{2+}+H_{2}O_{2}\to {\mathrm{Co}}^{3+}+{\mathrm{HO}}^{•}+{\mathrm{OH}}^{-}$$

The combination gives a high HO^•^ yield and rapid oxidative degradation, consistent with EPR and activity data. The enhancement via oxygen vacancies is also possible, and V_O_ might play a role as an electron donor, regenerating metal sites:

$$H_{2}O_{2}+V_{O}^{••}\to {\mathrm{HO}}^{•}+{\mathrm{OH}}^{-}$$

The absence of significant Fe edge changes confirms that iron cations in this case are not the primary redox centers and mainly provide the spinel lattice. Even though Fe^3+^ can be a Lewis acid, in these samples, its local coordination environment makes it less accessible for redox cycling with H_2_O_2_, so it does not play the main catalytic role.

### 3.9 Reuse and Stability

The sample Mn-3 has been investigated through cyclic experiments to assess its reusability and the variations in its activity over three consecutive cycles of CR and OTC degradation (Figure 10). The kinetic curves obtained from the UV–vis spectra for CR (Figure S15) and OTC (Figure S16) illustrate the degradation process for both compounds throughout the three cycles, as depicted in Figure 10a,10b, respectively.

**Figure 10:**
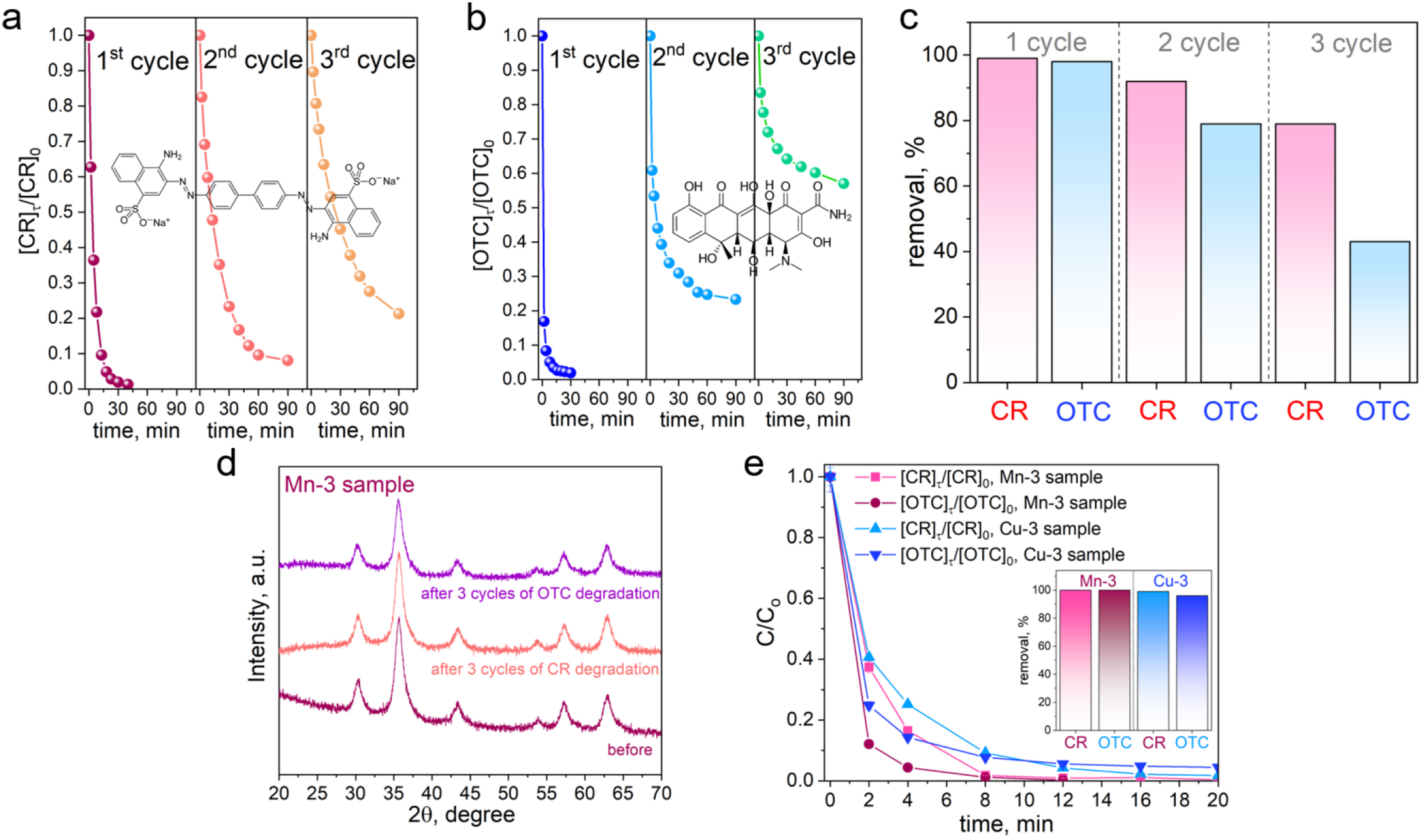
(a, b) Kinetic curves demonstrating the catalytic degradation of (a) Congo Red and (b) oxytetracycline using the Mn-3 sample and H_2_O_2_ during three cycles. (c) Removal (in %) of CR and OTC during the three cycles. Conditions: [CR] = 20 mg/L, [OTC] = 20 mg/L, *V* = 25 mL, [catalyst] = 1 g/L, [H_2_O_2_] = 10 mM, pH ∼ 7, *T* = 20 °C. (d) XRD patterns of the Mn-3 sample, obtained after three cycles. (e) Kinetic curves demonstrating the catalytic degradation of the OTC and CR mixture (inset: removal of CR and OTC in %) (conditions: [CR] = 10 mg/L, [OTC] = 10 mg/L, *V* = 25 mL, *m* = 25 mg, [H_2_O_2_] = 10 mM, pH ∼ 7, *T* = 20 °C).

The characteristic absorbance peak of CR (at λ = 498 nm) gradually decreases, indicating the progressive breakdown of the dye molecule (Figure S15). The green spectrum at 40 min confirms near-complete decolorization, suggesting efficient degradation of CR during the first cycle. The catalyst exhibits rapid reaction initiation within 13 min, leading to almost complete removal within 40 min. The results indicate that Mn-3 exhibits sufficient catalytic activity during all three cycles of CR degradation. In contrast, its efficiency in OTC degradation is notably effective only during the first two cycles (Figures 10b and S16). Specifically, the CR removal decreased from 99% to 92% in the second cycle. In contrast, the degradation of OTC decreased from 98% to 79% (Figure 10c). In the third cycle, CR removal remains acceptable (79%); however, OTC degradation drops significantly to 43%. This degradation trend confirms that while the catalyst retains activity for CR, it is less reusable for OTC, possibly due to stronger adsorption of OTC intermediates or higher catalyst surface passivation. This also suggests different surface interactions or degradation pathways between the two pollutants. This reduction in catalytic activity can also be attributed to surface inactivation caused by the chemisorption of pollutants onto the catalyst’s surface. Notably, the XRD analysis confirms that the spinel structure of the catalyst remains unchanged (Figure 10d).

The Cu-3 sample was also studied in cyclic experiments to evaluate its activity toward CR (Figure S17a) and OTC degradation (Figure S17b). It is obvious that the activity of the Cu-3 sample is lower than that of the Mn-3 sample. In addition, the removal efficiency of the Cu-3 sample toward CR is higher than toward OTC. During the first cycle, the Cu-3 sample removes 95% of CR within 60 min, whereas in the second cycle, it removes 90% of CR after 180 min (Figure S17c). This decrease in activity can be explained by saturation of the Cu-3 surface by chemisorbed CR molecules. The activity toward OTC removal is lower, indicating that chemisorption of OTC molecules is stronger and leads to surface deactivation. During the first cycle, the Cu-3 sample removes 90% of OTC within 80 min, whereas in the second cycle, it removes only 61% within 120 min (Figure S17b). The XRD patterns of the Cu-3 samples obtained before and after the removal processes (Figure S17d) demonstrate the stability and preservation of the spinel structure. The morphologies of the Mn-3 and Cu-3 samples after the removal processes, obtained by SEM, are shown in Figure S18a,b. The particles retain their spherical morphology, and no visible changes in surface structure are observed.

Figure 10e illustrates the capability of the Mn-3 and Cu-3 catalysts to simultaneously degrade a mixture of CR and OTC (10 mg/L concentration each). The degradation was monitored by UV–vis spectroscopy, accounting for the different absorption maxima of the two pollutants: 345 nm for OTC and 498 nm for CR (as shown in Figure S19a,b). The kinetics of their degradation are shown in Figure 10e. Notably, the Mn-3 sample exhibited impressive catalytic performance, achieving complete degradation of both pollutants in just 12 min, while the Cu-3 catalyst followed closely, reaching a degradation efficiency of 98% within 20 min. This is highlighted in the inset of Figure 10e, which confirms the effectiveness of both catalysts in eliminating approximately 100% of the CR and OTC mixture by Mn-3 and 98% by Cu-3. The faster degradation of OTC compared to CR using cobalt ferrite-based Fenton-like processes can be attributed to several mechanistic and physicochemical factors. First, OTC contains electron-rich functional groups, such as phenolic hydroxyls and amides, demonstrating high reactivity toward hydroxyl radicals. In contrast, the CR molecule has more chemically stable azo (−N = N−) and sulfonate groups, making it less susceptible to oxidative attack. Additionally, OTC exhibits stronger adsorption onto the catalyst surface via hydrogen bonding and π–π stacking with surface metal ions or hydroxyl groups, resulting in higher local OTC concentrations near reactive sites. In contrast, CR, as an anionic molecule, faces steric hindrance and electrostatic repulsion that reduce its affinity for the surface. This suggests that OTC undergoes inherently faster reaction kinetics. Moreover, the smaller and more flexible structure of OTC allows for more efficient diffusion into the catalyst’s mesopores, compared to the planar structure of CR. Thus, these factors clarify the enhanced removal performance of OTC over CR in Fenton-like processes.

## 4 Conclusions

In this study, CoFe_2_O_4_@Co_3_O_4_, (Mn,Co)­Fe_2_O_4_@Co_3_O_4_ and (Cu,Co)­Fe_2_O_4_@Co_3_O_4_ hollow spheres were successfully synthesized via a solvothermal method. The resulting materials exhibit a unique core–shell architecture comprising a crystalline ferrite core and a porous shell of Co_3_O_4_ nanosheets/nanoparticles, yielding in high surface areas and improved dispersion in water. The doped samples, particularly Mn-3, showed significantly enhanced performance in both adsorption and Fenton-like catalytic degradation of model pollutants, Congo Red and oxytetracycline. The synergistic effect of doping and hollow morphology improved surface charge, surface area, and the availability of redox-active sites, resulting in faster and more efficient pollutant removal. A broad range of characterization techniques (XRD, TEM, BET, XAS, XPS, Raman, and EPR) provided comprehensive insights into the material structure and catalytic mechanism. Taking into account XAS and XPS data before and after adsorption/catalysis it can be concluded that Fe remains essentially redox-inert in this case, acting mainly as a structural framework. Co, Mn, and Cu doping centers provide the main redox activity. Raman spectroscopy suggested the presence of structural distortions and oxygen vacancies, which contribute to enhanced catalytic activity. EPR studies revealed that Cu-3 primarily generates hydroxyl radicals, whereas Mn-3 favors the production of singlet oxygen. Reusability tests confirmed the material’s stability, especially for CR degradation, although some deactivation was observed for OTC after repeated cycles, likely due to partial surface passivation by intermediate species. Overall, the hollow ferrite spheres offer a promising bifunctional platform for efficient, recyclable adsorption-catalysis in water purification applications.

## Supplementary Material





## Data Availability

The data underlying this study are openly available in RODBUK repository at 10.57903/UJ/6UKVRL.

## Notes

Demonstration of the magnetic separation of the adsorbent. The video shows the dispersion of Mn-substituted cobalt ferrite particles in water, followed by their rapid separation from the suspension under an external magnet, illustrating the strong magnetic response and the ease of catalyst recovery/separation (Video S1) (MP4)
Additional experimental data and characterization results are provided to complement the main manuscript. These include structural, morphological, spectroscopic, textural, and catalytic analyses of Mn- and Cu-substituted ferrite samples. Specifically, FTIR, diffuse reflectance, XRD, XPS, XAS, and nitrogen adsorption/desorption analyses are presented, along with SEM, TEM, and EDS characterization. UV–vis spectroscopy of pollutant degradation processes and recyclability studies are also included, together with tabulated crystallographic, textural, and surface charge parameters (PDF)
