# Supplementary material for: Core–Shell Mn- and Cu-Doped CoFe2O4@Co3O4 Hollow Spheres with Dual Adsorption and Catalytic Function
Source: Inorg Chem. 2026 Jan 22;65(4):2307–25. doi: 10.1021/acs.inorgchem.5c04962 (PMC12869482; doi:10.1021/acs.inorgchem.5c04962)
Supplement: Supplementary file 2 [file ic5c04962_si_002.pdf]

Supporting Information

Core–Shell Mn- and Cu-Doped $CoFe_2O_4$@Co_3O_4 Hollow Spheres with Dual Adsorption and Catalytic Function

Tetiana Tatarchuk [a,b,c,*], Wojciech Macyk [a], Vitaliy Bilovol [d], Krystian Sokolowski [d], Marcin Sikora [e], Kamila Sobańska [a], Piotr Pietrzyk [a]

[a] *Faculty of Chemistry, Jagiellonian University, Gronostajowa str. 2, 30-387 Kraków, Poland*
[b] *Educational and Scientific Center of Materials Science and Nanotechnology, Vasyl Stefanyk Carpathian National University, 76-018 Ivano-Frankivsk, Ukraine*
[c] *Jerzy Haber Institute of Catalysis and Surface Chemistry Polish Academy of Sciences, Niezapominajek str. 8, 30-239 Kraków, Poland*
[d] *Academic Centre for Materials and Nanotechnology, AGH University of Krakow, Av. Mickiewicza 30, 30-059 Kraków, Poland*
[e] *National Synchrotron Radiation Centre SOLARIS, Jagiellonian University, Czerwone Maki str. 98, 30-392 Kraków, Poland*

** corresponding author: Tetiana Tatarchuk, tetiana.tatarchuk@uj.edu.pl*

Table of content:

Text S1. Characterizations and analysis methods.

Text S2. Energy-dispersive spectroscopy (EDS).

Text S3. FTIR analysis of as-synthesized and annealed Mn- and Cu-containing samples.

Text S4. Diffuse reflectance spectroscopy.

Text S5. N_2 adsorption/desorption isotherm analysis.

Text S6. FTIR spectroscopy of the sample after OTC adsorption/catalytic degradation.

Text S7. FTIR spectroscopy of the sample after CR adsorption/catalytic degradation.

Figure S1. XRD patterns of Mn- and Cu-containing cobalt ferrites, obtained by the solvothermal method: (a) as-synthesized precursors and (b) samples, annealed at 300 °C.

Figure S2. (a) EDS spectra and (b) SEM image of CFO sample and the (c) corresponding elemental mapping for (d) Co, Fe, and O. (e) EDS spectra and (f) SEM image of Mn-3 sample and the (g) corresponding elemental mapping for (h) Co, Fe, Mn, and O. (i) EDS spectra and (j) SEM image of Cu-1 sample and the (k) corresponding elemental mapping for (l) Co, Fe, Cu and O.

Figure S3. (a) TEM image of the Mn-1 sample; (b) TEM image of the Mn-3 sample; (c) TEM image of the Cu-1 sample; (d) HR-TEM image of Mn-3 sample annealed at 400°C.

Figure S4. FT-IR spectra of (a) as-synthesised and annealed at (b) 300°C and (c) 400°C Mn- and Cu-

containing cobalt ferrites.

Figure S5. XAS at the Fe L_2-edge normalized to the intensity of the peak at 723 eV of all the samples.

Figure S6. Diffuse reflectance spectroscopy: (a) The optical absorption spectra of Mn-doped cobalt ferrites. (b) UV-Vis diffuse reflectance spectra of the samples transformed to the Kubelka-Munk function. (c) Plots of $[F(R)h\nu]^{1/2}$ *vs.* [hν].

Figure S7. (a) The fit of the Mn-3 sample and the (b) corresponding magnetic hyperfine distribution P(B)-curves derived from the spectrum.

Figure S8. (a) N_2 adsorption/desorption isotherms and (b) pore size distributions for ferrite samples annealed at 300°C.

Figure S9. (a) ξ-potential. (b-d) SEM photos of the most representative (b) CFO, (c) Mn-3, and (d) Cu-1 samples.

Figure S10. (a) UV-Vis spectra of OTC and OTC+H_2O_2 mixture, taken at 1 min, 30 min, 1 hour, and 2 hours. (b) UV-Vis spectra of CR and CR+H_2O_2 mixture, taken at time intervals of 8 hours and 72 hours.

Figure S11. The initial rate of (a) OTC and (b) CR removal normalized to the specific surface area of the samples.

Figure S12. Survey XPS spectra of model OTC (a) and CR (b) pollutants and Mn-3 and Cu-3 ferrites before and after OTC (a) and CR (b) adsorption and catalysis removal.

Figure S13. High-resolution XPS spectra of (a) Co $2p_{3/2}$, (b) Fe $2p_{3/2}$, (c) Mn $2p_{3/2}$, and (d) Cu $2p_{3/2}$, for Mn-3 and Cu-3 samples before and after CR/OTC adsorption/catalysis.

Figure S14. High-resolution XPS spectra of (a) O 1s, (b) C 1s, (c) N 1s, and (d) S 2p for CR dye, OTC powder, and Mn-3, Cu-3 samples before and after CR and OTC adsorption and catalysis.

Figure S15. UV-Vis spectra of Congo Red during three consecutive degradation cycles using the Mn-3 sample. Conditions: [CR] = 20 mg/L, V = 25 mL, [Mn-3] = 1 g/L, [H_2O_2] = 10 mM, pH ~ 7, T = 20°C.

Figure S16. UV-Vis spectra of oxytetracycline during three consecutive degradation cycles using the Mn-3 sample. Conditions: [OTC] = 20 mg/L, V = 25 mL, [Mn-3] = 1 g/L, [H_2O_2] = 10 mM, pH ~ 7, T = 20°C.

Figure S17. (a,b) Kinetic curves demonstrating the catalytic degradation of the (a) Congo Red and (b) oxytetracycline using the Cu-3 sample and H_2O_2 during three cycles. (c) Removal (in %) of CR and OTC during the three cycles. Conditions: [CR] = 20 mg/L, [OTC] = 20 mg/L, V = 25 mL, [catalyst] = 1 g/L, [H_2O_2] = 10 mM, pH ~ 7, T = 20°C. (d) XRD patterns of the Mn-3 sample, obtained after three cycles.

Figure S18. SEM images of (a) Mn-3 and (b) Cu-3 samples after catalysis.

Figure S19. UV-Vis spectra of the mixture of Congo red and oxytetracycline decomposed using (a)

Mn-3 and (b) Cu-3 samples. Conditions: [CR] = 10 mg/L, [OTC] = 10 mg/L, V = 25 mL, [catalyst] = 1 g/L, [H_2O_2]= 10 mM, pH ~ 7, T = 20°C.

Table S1. Summary of interplanar spacings and lattice parameters from XRD analysis.

Table S2. Textural characteristics of Mn- and Cu-containing cobalt ferrites, annealed at 300°C.

Table S3. Textural characteristics of Mn- and Cu-containing cobalt ferrites, annealed at 400°C.

Table S4. Hydrodynamic size of particles, polydispersity index (PDI), and surface charge of Mn- and Cu-containing ferrites annealed at 400°C.

References

Text S1. Characterizations and analysis methods

The X-ray diffraction (XRD) patterns were recorded on a Rigaku MiniFlex 600 diffractometer using Cu K_{α} radiation at a wavelength of 1.5406 Å. The scan step was 0.02° (2θ), and the scan rate was 1°/min.

The morphology of the samples was examined using a Helios 5 Hydra DualBeam scanning electron microscope (SEM). The samples were deposited on carbon tape without gold coating.

ATR-FTIR spectra were recorded using a Thermo Scientific Nicolet Summit FTIR spectrometer with a diamond window and the OMNIC Paradigm Software (Thermo Fisher Scientific, Waltham, Massachusetts, USA). The spectra were recorded in the wavenumber range 4000–400 cm^{-1} with a resolution of 4 cm^{-1} and 64 scans.

Diffuse reflectance spectra were recorded using a Shimadzu UV-3600 spectrophotometer equipped with an integrating sphere with a diameter of 15 cm. The tested samples were ground with $BaSO_4$ (1:50) and pressed into pellets. Pure barium sulfate was used as a reference material. The Kubelka-Munk function F(R) was calculated as follows: $F(R)=(1–R)^2/2R$, where R is the measured value of the reflectance. The Tauc plot was obtained by plotting $[F(R)\cdot h\nu]^{1/2}$ against $h\nu$, where $h\nu$ is the photon energy. The exponent is ½ for the case of an indirect allowed transition. Tauc plots baselines were corrected by linear fitting.

The specific surface area and pore size distribution of the catalysts were determined using N_2 adsorption/desorption isotherms at –196°C (Autosorb iQ-MP, Quantachrome instruments). The samples were outgassed under vacuum at 300°C for 24 hours before the analysis. The Braunauer-Emmett-Teller equation was used to calculate the specific surface area.

Transmission electron microscopy (TEM) imaging was performed using a Tecnai Osiris instrument (FEI) with an X-FEG Schottky field emitter, operating at an accelerating voltage of 200 kV and a Rio16 camera. The samples were deposited on a lacey carbon film supported on a copper grid (Agar Scientific, 400 mesh).

The hydrodynamic diameter, polydispersity index (PDI), and zeta potential of the synthesized hollow spheres were measured using a Zetasizer Nano (Malvern Instruments) equipped with a 633 nm laser. For DLS analysis, the samples were dispersed in deionized water using ultrasonic treatment for 10 minutes to ensure uniform dispersion and reduce aggregation. Measurements were performed at room temperature, and each sample was analyzed in triplicate. The hydrodynamic size and PDI were calculated based on intensity-weighted distributions. Zeta potential measurements were also carried out in deionized water using the same instrument in electrophoretic light scattering mode. All values represent the average of three measurements.

Raman measurements were performed on a Renishaw InVia spectrometer coupled to a Leica optical microscope. The radiation source was a red laser with a wavelength of 785 nm. Spectra were measured in the range of 100–1400 cm^{-1}, and a 50× magnification objective was used.

The ^{57}Fe Mössbauer spectra of the samples were recorded in transmission mode at room temperature using a ^{57}Co source in a Rh matrix, emitting 14.4 keV γ-rays. The measurements were performed using an electromechanical RENON MsAa-4 spectrometer operating in triangular mode, equipped with a krypton-filled proportional detector. Spectra were recorded using a 1024-channel analyzer. The spectra were analyzed using a least-squares fitting procedure.

Magnetic measurements were carried out at room temperature using a vibrating sample magnetometer (Lakeshore 7400) under an applied magnetic field of up to 1.6 T.

Near-edge X-ray absorption fine structure at Fe, Co, Mn, and Cu $L_{3,2}$ absorption edges was collected at room temperature at the XAS end-station of the PIRX (04 BM) beamline at SOLARIS synchrotron (Krakow, Poland). X-ray photons were monochromatized by a plane grating with 800 lines/mm and an exit slit opened to 50 µm, which provides a resolving power of $E/\Delta E \sim 4000$. Nanopowders were probed under UHV conditions by being attached to an Omicron flag-style sample holder using conductive carbon tape (Agar Scientific Ltd). Data were collected using drain current as a probe of total electron yield sensitive to the surface structure of a typical thickness of 5–10 nm.

XPS measurements were performed with a PHI VersaProbe II apparatus. The samples were irradiated with a focused monochromatic Al Kα (E = 1486.6 eV) X-ray beam with a diameter of 100 μm, and the beam was rastered over an area of 400 × 400 μm^2. The pass energy of the analyzer was set to 117.50 eV (0.5 eV step) for survey scans and 46.95 eV (0.1 eV step) for high-energy resolution spectra. A dual beam charge neutralization with 1 eV electrons and low-energy (7 eV) monoatomic Ar^+ ions was used to avoid charging effects. The spectra were referenced to the neutral (C–C) carbon C 1s peak, with a binding energy of 285.0 eV. Deconvolution of spectra was carried out using PHI MultiPak software (v.9.9.3).

Text S2. Energy-dispersive spectroscopy (EDS).

EDS was used for the quantitative analysis of the chemical composition of the studied samples. Representative results for the most characteristic samples are presented in Fig. S2. Taking into account the mass percentage of each element, the molar ratio has been calculated. In the case of CFO, Mn-1, and Mn-3 samples, the molar ratio of Co/Mn/Fe was calculated as 0.82/0.0/2, 0.53/0.04/2, and 0.42/0.03/2, respectively, which differ slightly from the expected ones. For Cu-containing samples, the elemental mapping analysis revealed a uniform distribution of Co, Fe, Cu, and O within the microspheres. For Cu-1 and Cu-3 samples, the molar ratios of Co:Cu:Fe were calculated as 0.6/0.2/2 and 0.7/0.2/2, respectively. These results align with the nominal values only for the Cu-3 (0.7/0.3/2) sample. Nevertheless, the Cu-1 sample exhibits a deviation in elemental composition from the nominal values (0.9/0.1/2), with a lower amount of Co ions and a higher amount of Cu than the nominal ratio. It is plausible that the shell did not achieve complete surface coverage or may have been inadequately thin, which could have attenuated the anticipated Co/Cu/Mn signal due to the predominance of the signal from the Fe-rich core. Additionally, it is possible that during the synthesis process, some cations were not fully incorporated into the shell structure. Furthermore, from a thermodynamic perspective, iron dominates spinel formation, potentially displacing cobalt and manganese in the process. In addition, some amount of Co_3O_4 may form in such conditions due to the decomposition of Co glycerolate [1].

Text S3. FTIR analysis of as-synthesized and annealed Mn- and Cu-containing samples.

FTIR spectroscopy has been utilized as a tool for identifying functional groups in the samples' structure. As shown in Fig. S4a, the spectra of the as-synthesized samples contain multiple peaks and differ significantly from the spectra of annealed ones (Figs. S4b,c). The wide band centered at 3330-3340 cm^{-1} is responsible for hydrogen-bound hydroxyl groups [2,3]. The sharp band centered at 1608-1620 cm^{-1} is characteristic of the δ_{H_2O} bending signal [2,4]. It becomes wider with the increasing Mn or Cu content. A similar correlation is observed with the peak at 3340 cm^{-1}, which also becomes wider for the doped samples. A clearly defined peak at 1610 cm^{-1} in the precursors' spectra, peculiar to bending vibrations of water molecules, almost disappeared after the sample annealing. Only very weak bands shifted to 1630 and 1644 cm^{-1} can be seen in the spectra of samples annealed at 300 and 400°C, respectively. The bands at 1300-1400 cm^{-1} are assigned to C–H bending [3,5]. This band becomes more intense with an increase in Mn content. C–O and C–C stretching vibrations are observed in the range of 1047-1120 cm^{-1} and 810-820 cm^{-1}, respectively [3]. The strong absorption bands in the 1260-1419 cm^{-1} region correspond to NO_3^- ions [6], originating from the metal salts used for the synthesis. This band is weakly represented at around 1340 cm^{-1} in annealed at 300°C samples and disappears completely in the samples annealed at 400°C. The group of small bands in the region from 990 to 1170 cm^{-1} is related to C–O stretching vibration [7], while the absorption band at 809 cm^{-1} in precursors can be attributed to C–H bending vibration [7]. The peaks observed in the spectral region between 350 and 600 cm^{-1} are attributed to the vibrations of the metal-oxygen (M–O) bonds within the tetrahedral and octahedral sublattices of the spinel structure [8]. These characteristic peaks in all samples signify the initiation of spinel nucleation during the precursor synthesis stage. Notably, this observation aligns with the data obtained from XRD analysis, providing further support for the spinel formation process at the precursor stage. More precisely, the peaks, corresponding to A-sites, are observed at 580, 560, and 530 cm^{-1} for as-synthesised samples, whereas the peaks, corresponding to B-sites, are observed at 484, 487, and 491 cm^{-1}. The peaks shifting testify to the introduction of Mn ions into the crystal structure.

The thermal treatment of the samples at 300°C and 400°C changes the FTIR spectra (Fig. S4b,c). The weak signal at 3300 cm^{-1} remained, indicating the presence of structurally bound OH– groups. The peaks attributed to the vibrations of the metal-oxygen (M–O) bonds become more intense, which can be attributed to the improved crystallinity of the spinel structure caused by thermal treatment. The calcination weakened the C–H bending and C–C stretching vibrations, indicating the almost complete removal of organic groups from all samples. The observed double peak at 2360 cm^{-1} can be attributed to surface-adsorbed CO_2. Notably, the introduction of Mn and Cu ions in the spinel structure leads to an increase in the intensity of the CO_2 peak, indicating the enhanced ability of manganese/copper ions to bond with carbon dioxide molecules. It was noted that this band, signifying the presence of carbonate species, persists in the samples subjected to annealing at 300°C. However, in the samples annealed at 400°C, this peak is observed in the samples containing Mn/Cu. This could be evidence of acid-base surface properties: the higher the Mn/Cu loading, the more basic surface centers [9].

Text S4. Diffuse reflectance spectroscopy.

Figure S6a presents the UV-Vis absorption spectra of Mn- and Cu-doped cobalt ferrites at room temperature in the 200-2100 nm range. The samples reveal absorption bands within the UV-vis and NIR regions, pointing to their rich redox and photo-redox properties. The Kubelka-Munk theory, which combines the reflectance and absorption coefficient, was applied for spectra analysis (Figure S6b): $F(R) = \alpha/s = (1–R)^2/2R$, where F(R) is the Kubelka–Munk function, R is the reflectance, α is the absorption coefficient, and s is the scattering factor. Since the scattering factor is only slightly wavelength dependent, F(R) is proportional to α [10]. The band gap for synthesised samples has been calculated using the Tauc plot $[F(R)\cdot h\nu]^{n/2}$ *vs.* hν (n = 1 and 4 for indirect and direct allowed transitions, respectively) by extrapolating the slope to $[F(R)\cdot h\nu]^{n/2} \rightarrow 0$ as illustrated in Figure S6c. Based on the data previously reported in the literature, the cobalt ferrites demonstrate the indirect energy gap. Mn and Cu doping slightly reduces the band gap value from 1.30 eV (for CFO) to 1.29 eV (for Mn-3, Cu-1, and Cu-3 samples).

Text S5. N_2 adsorption/desorption isotherm analysis.

The textural properties of the samples were evaluated using N_2 physisorption isotherms and BJH pore size distribution curves (Figs. S8 and 5a,b; Tables S2 and S3). The BET surface area is affected by both Mn and Cu content and annealing temperature: the primary introduction of Mn or Cu ions into cobalt ferrite does not change significantly the BET surface area of samples annealed at 300°C (S_{BET}(CFO-300) = 100.2 m^2/g, S_{BET}(Mn-1-300) = 110.1 m^2/g, S_{BET}(Cu-1-300) = 98.9 m^2/g), while the further increase in Mn or Cu content increases S_{BET} up to 159.8 m^2/g (Mn-3-300) and 118.41 m^2/g (Cu-3-300). As expected, the annealing at 400°C decreased the S_{BET} of the samples and placed them in the order: S_{BET}(Mn-3) = 115.1 m^2/g > S_{BET}(Mn-1) = 80.7 m^2/g > S_{BET}(Cu-3) = 75.4 m^2/g > S_{BET}(CFO) = 58.9 m^2/g > S_{BET}(Cu-1) = 49.5 m^2/g.

Annealing at 300°C results also in the formation of large mesopores in the range of 1.5-30 nm with a total volume of 0.3240, 0.2153, 0.2693, 0.2452, and 0.2652 $cm^3 \cdot g^{-1}$ for CFO-300, Mn-1-300, Mn-3-300, Cu-1-300, and Cu-3-300 samples, respectively (Fig. S8b, Table S2). The average pore diameter is 13 nm for cobalt ferrite and 7 nm for the Mn-3-300 sample. Introducing Mn or Cu ions into the spinel structure results in enhanced morphology and improved mesoporous structure.

The pore size distribution within the 1-100 nm range was assessed utilising the BJH method applied to the adsorption branch of the isotherm (Fig. S8b). This approach was chosen for its superior reliability compared to analysing the desorption branch [11]. A typical mesoporous metal oxide offers pores of only 3-4 nm [11]. This study found smaller mesopores and higher values of specific surface area in Mn-containing samples. The predominance of 3 nm mesopores in the Mn-3-300 sample indicates that this sample has the highest specific surface area of 159.8 m^2/g compared to cobalt ferrite (S_{BET} = 100.2 m^2/g) and Mn-1-300 (S_{BET} = 110.1 m^2/g). For Cu-containing samples, the diameters of mesopores range between 1.5-30 nm (in the case of Cu-1-300 samples) and 3.5-30 nm (for Cu-3-300 sample) (Fig. S8b). Upon closer inspection, the PSD curves of the CFO-300 and Cu-1-300 samples are similar, with a peak at 3.5 nm. Conversely, the PSD curve of the Cu-3-300 sample demonstrates a shift towards larger mesopores, with a peak centred at 5.5 nm.

Text S6. FTIR spectroscopy of the sample after OTC adsorption/catalytic degradation.

FTIR spectra of the most active samples, Mn-3 and Cu-3, before and after OTC adsorption/catalytic degradation are presented in Figure 6g,h. The peak at 1636 cm^{-1} is typically attributed to the O–H bending of adsorbed water or surface OH-groups. After the adsorption of OTC, a shift to ~1600 cm^{-1} is noted for both active samples, Mn-3 and Cu-3, which indicates a significant interaction between the OTC molecules and the catalysts' surface. The increase in width and intensity of this peak suggests overlapping with additional peaks, likely with the peak around 1600 cm^{-1}, which can be attributed to the stretching of C=C or C=O present in OTC structures. This observation suggests the possible formation of hydrogen bonds or coordination bonds between OTC functional groups (C=O or $–NH_2$) and the metal centres on the catalyst surface. Such interactions imply a chemisorption mechanism, which is crucial for catalytic activity. For the Mn-3 sample, the FTIR peak at ~542 cm^{-1}, attributed to M–O stretching vibration in the ferrite structure, shifted to 537 cm^{-1} after OTC adsorption, indicating the interaction (probably via coordination) between the antibiotic molecule and metal centres. Interestingly, after the catalytic process, the peak is observed at 543 cm^{-1}, suggesting that the interaction is reversible and returns to its initial position, meaning that the catalyst structure is preserved. For the Cu-3 sample, the peak at 529 cm^{-1} shifted to 533 cm^{-1} after OTC adsorption and catalytic degradation. This shift is likely due to surface complexation and redox-induced M–O bond changes, specifically involving Cu–O and Co–O bonds. OTC not only adsorbs on the surface but also interacts chemically with active metal centres, modifying the local bonding environment to some extent.

Another contributing factor is the presence of H_2O_2 during the catalytic stage, which can promote redox cycling between oxidation states (e.g., Cu^{2+}/Cu^{+} or Co^{3+}/Co^{2+}). These redox transitions modify the local electronic environment, affecting the strength of the M–O bonds. Specifically, oxidation tends to strengthen M–O bonds, increasing their vibrational frequency. This is observed in the FTIR spectrum as a shift to higher wavenumbers, for example, from 529 to 533 cm^{-1}. The FTIR band at 1473 cm^{-1}, attributed to C–H bending vibrations from CH_2/CH_3 groups, was shifted to 1456

cm^{-1} after oxytetracycline was adsorbed. This shift suggests that the non-polar or aromatic parts of the OTC molecule interact with the ferrite surface through a multi-point adsorption mechanism, where various interactions enhance the bonding between OTC and the catalyst. After the adsorption of oxytetracycline, new FTIR peaks were observed at 1258-1259, 1220, and 1168 cm^{-1}. These peaks correspond to C–N stretching, phenolic C–O stretching, and C–O/C–H vibrations, respectively, confirming OTC adsorption onto the ferrite surface. The interactions likely involve hydroxyl, amide, and aromatic groups, highlighting the significant role of these functional groups in the adsorption. The spectrum of a sample participating in the catalytic degradation process shows a notable reduction in the OTC-associated FTIR peaks at 1259, 1220, and 1168 cm^{-1}, confirming the degradation of key functional groups in OTC. Concluding, the catalytic degradation of OTC was effective, and the catalyst surface remained relatively clean, with no significant fouling from residual by-products.

Text S7. FTIR spectroscopy of the sample after CR adsorption/catalytic degradation.

The adsorption of Congo Red onto the Cu- and Mn-containing ferrites was further confirmed by FTIR spectroscopy (Figs. 7g,h). After the adsorption step, several characteristic peaks corresponding to the CR structure appeared/shifted in the spectra of the used materials, indicating successful interaction with the dye molecules. For example, the azo (–N=N–) stretching vibration, typically observed at around 1580 cm^{-1}, shifted to 1559 cm^{-1}, suggesting possible coordination with surface metal centres. The strong band observed at 1044 cm^{-1} is attributed to the sulfonate groups $–SO_3^-$, indicating electrostatic interactions between the negatively charged sulfonate groups and the positively charged surface sites of the ferrites. The intensity of this band is twice as low after catalysis, indicating the degradation of the CR molecule using H_2O_2 and ferrite catalyst. In addition, aromatic C=C stretching vibrations (1450-1600 cm^{-1}) could be attributed to interactions between the aromatic rings of the CR molecule and the ferrite surface. The shift of the FTIR peak from 541 to 544 cm^{-1} after CR degradation in the Mn-3 sample (Figure 7g) suggests changes in the M–O bonding environment, likely due to surface complexation and redox effects involving Mn–O and Co–O bonds. This indicates that CR interacts chemically with active metal sites. The presence of H_2O_2 promotes redox cycling (e.g., Mn^{3+}/Mn^{2+}, Co^{3+}/Co^{2+}) that alters the electronic structure and strengthens M–O bonds, leading to a higher vibrational frequency. For the Cu-3 sample, the FTIR band attributed to M–O vibrations appeared at 530 cm^{-1} in the starting material (Figure 7h) . After Congo Red adsorption, this band shifted to 537 cm^{-1}, indicating surface interactions between the dye molecules and the M–O species, likely involving electrostatic attraction or surface complexation with Cu–O and Co–O sites. Interestingly, the band remains at its original 530 cm^{-1} position after catalytic degradation, despite FTIR evidence of residual CR on the surface. While CR molecules or the degradation intermediates remain adsorbed, they no longer significantly perturb the local M–O bonding environment. One possible explanation is that these remaining species are weakly bound and do not cause a measurable shift in vibrational frequencies. This also implies that the catalyst retains structural stability during the Fenton-like conditions, and the core M–O bonds are not chemically altered by the presence of

residual organics. It can be concluded that the hollow structure and surface chemistry (due to Mn or Cu doping) greatly enhance CR and OTC adsorption, likely due to the increased surface area, surface charge interactions, and coordination between pollutant's functional groups (like amines or ketones) and metal ions (Mn^{2+}, Cu^{2+}).

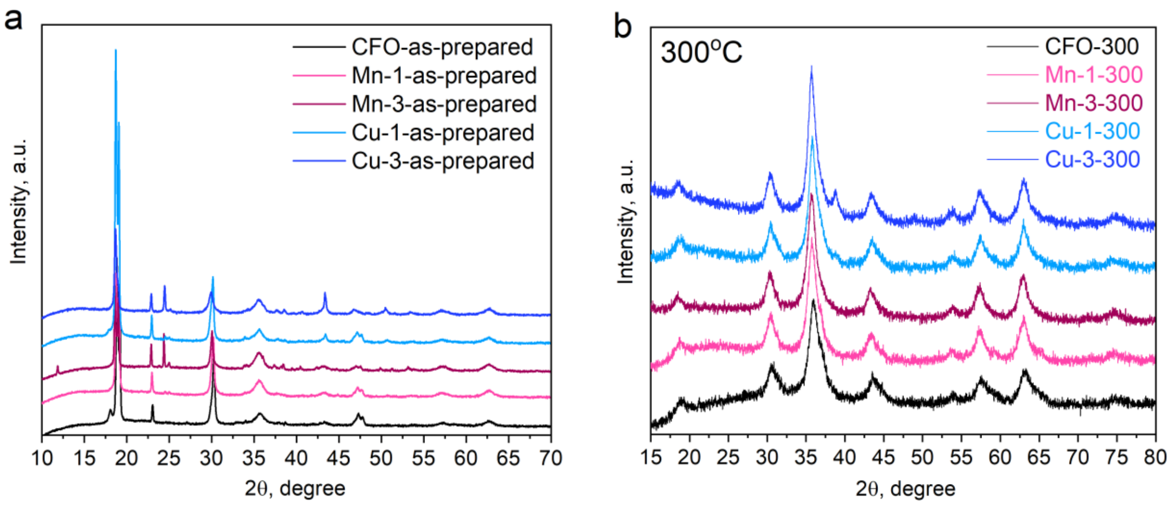

Figure S1. XRD patterns of Mn- and Cu-containing cobalt ferrites, obtained by the solvothermal method: (a) as-synthesized precursors and (b) samples, annealed at 300 °C.

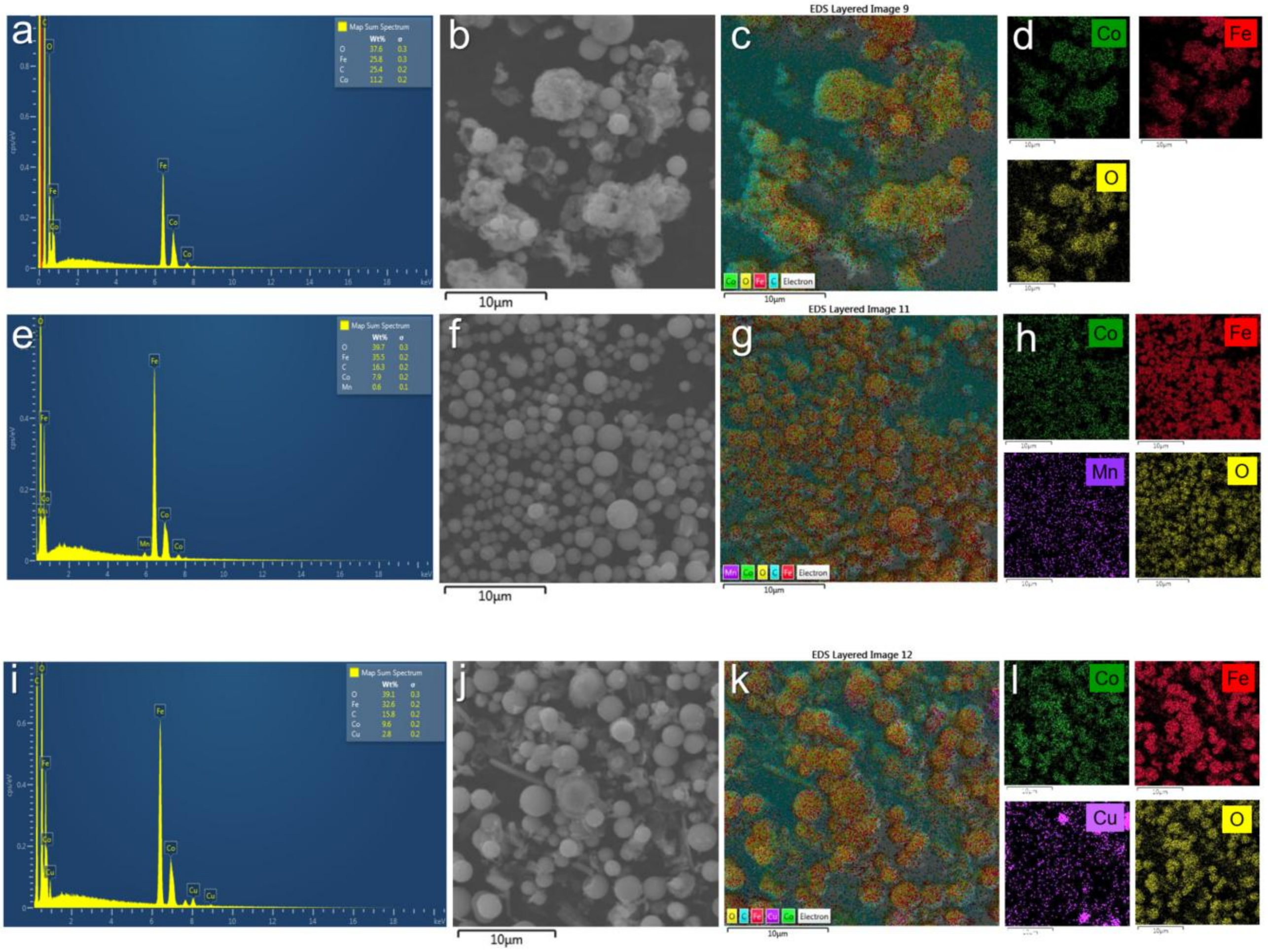

Figure S2. (a) EDS spectra and (b) SEM image of CFO sample and the (c) corresponding elemental mapping for (d) Co, Fe, and O. (e) EDS spectra and (f) SEM image of Mn-3 sample and the (g) corresponding elemental mapping for (h) Co, Fe, Mn, and O. (i) EDS spectra and (j) SEM image of Cu-1 sample and the (k) corresponding elemental mapping for (l) Co, Fe, Cu and O.

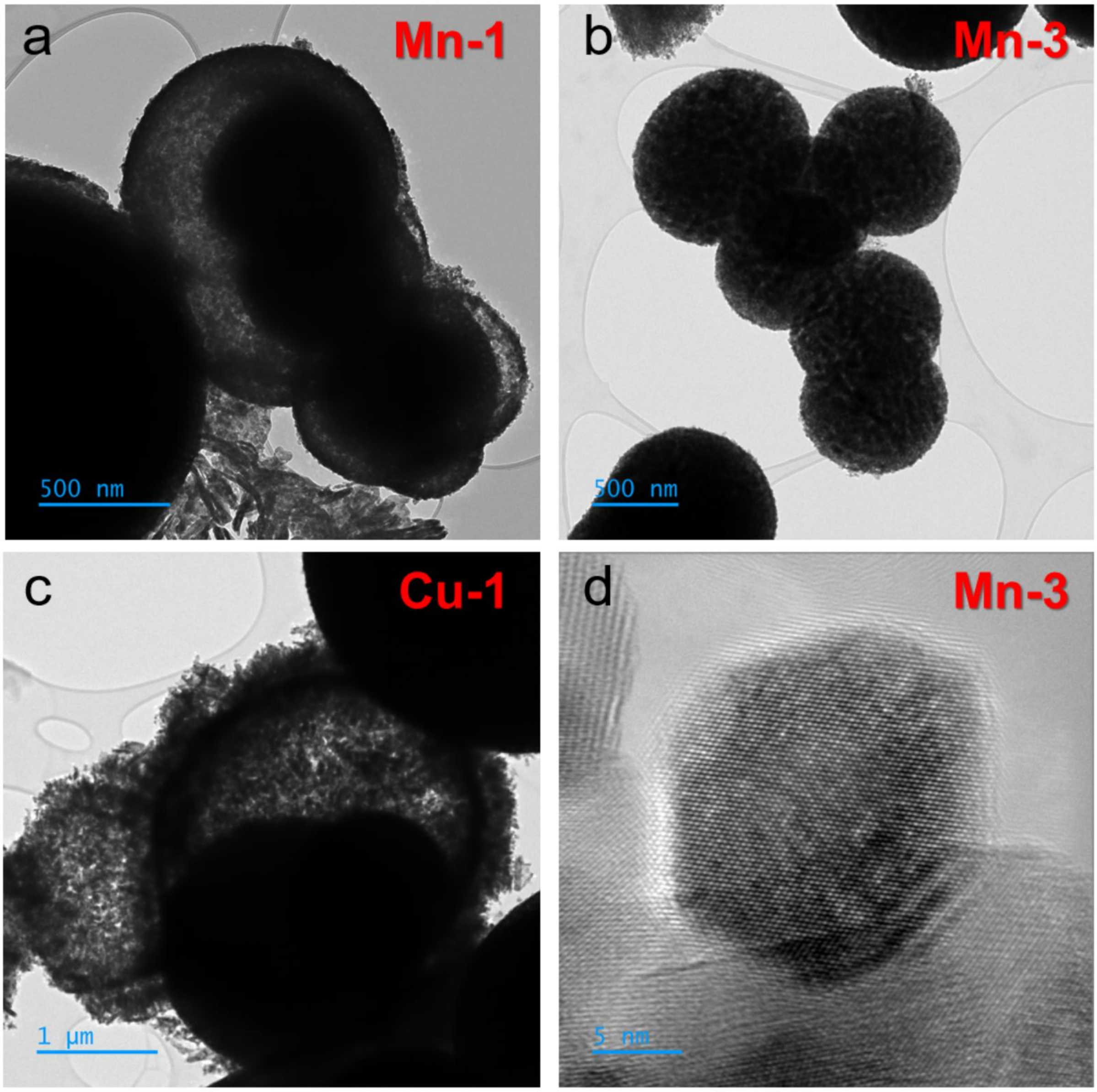

Figure S3. (a) TEM image of the Mn-1 sample; (b) TEM image of the Mn-3 sample; (c) TEM image of the Cu-1 sample; (d) HR-TEM image of Mn-3 sample annealed at 400°C.

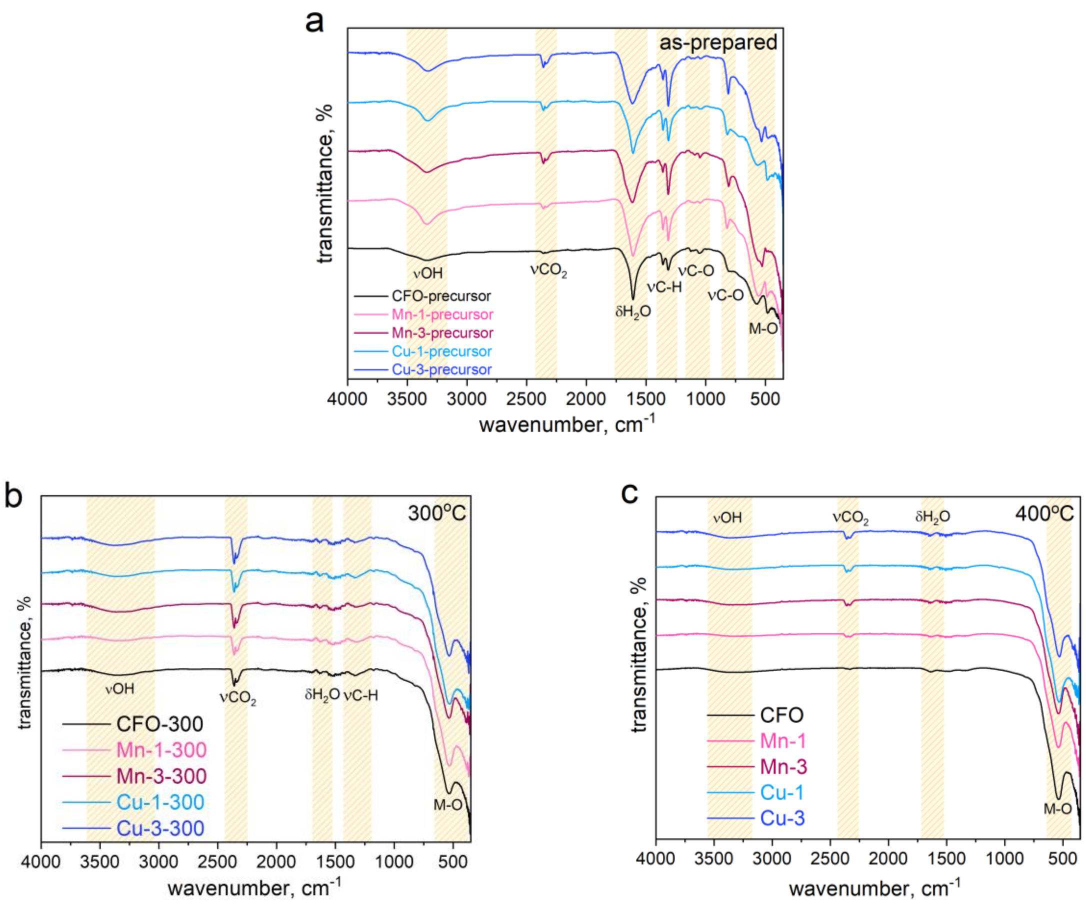

Figure S4. FT-IR spectra of (a) as-synthesised and annealed at (b) 300°C and (c) 400°C Mn- and Cu-containing cobalt ferrites.

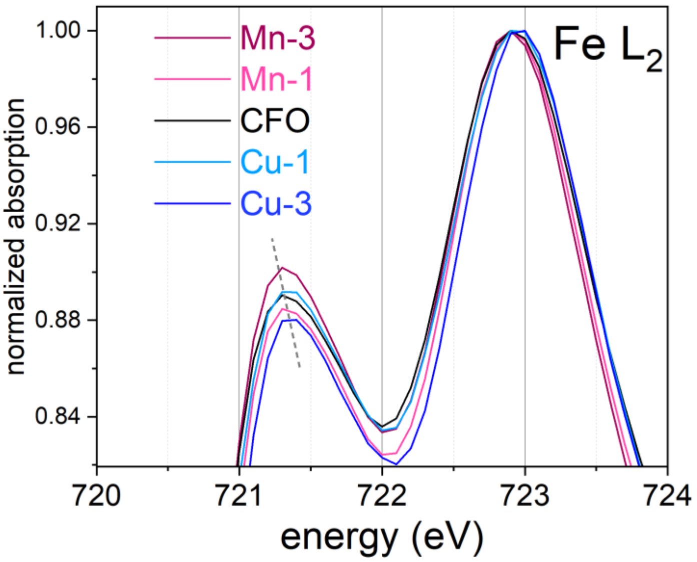

Figure S5. XAS at the Fe L_2-edge normalized to the intensity of the peak at 723 eV of all the samples.

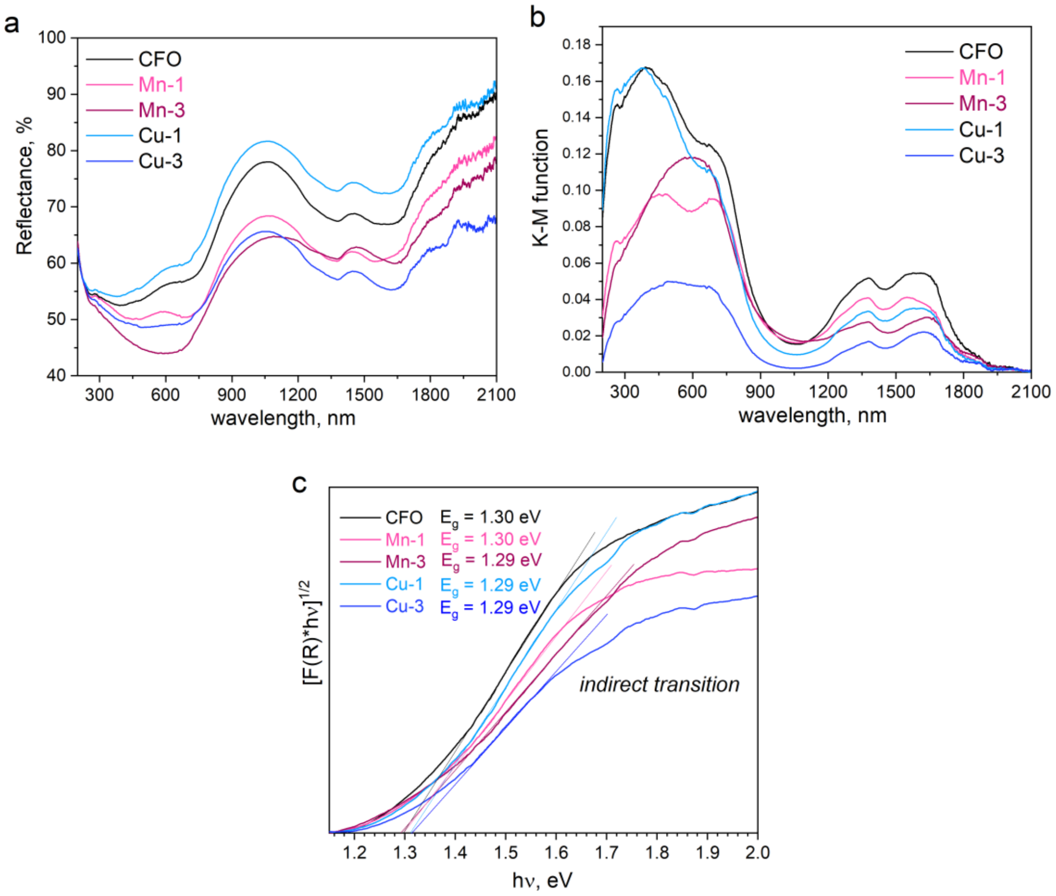

Figure S6. Diffuse reflectance spectroscopy: (a) The optical absorption spectra of Mn-doped cobalt ferrites. (b) UV-Vis diffuse reflectance spectra of the samples transformed to the Kubelka-Munk function. (c) Plots of $[F(R)h\nu]^{1/2}$ *vs.* $[h\nu]$.

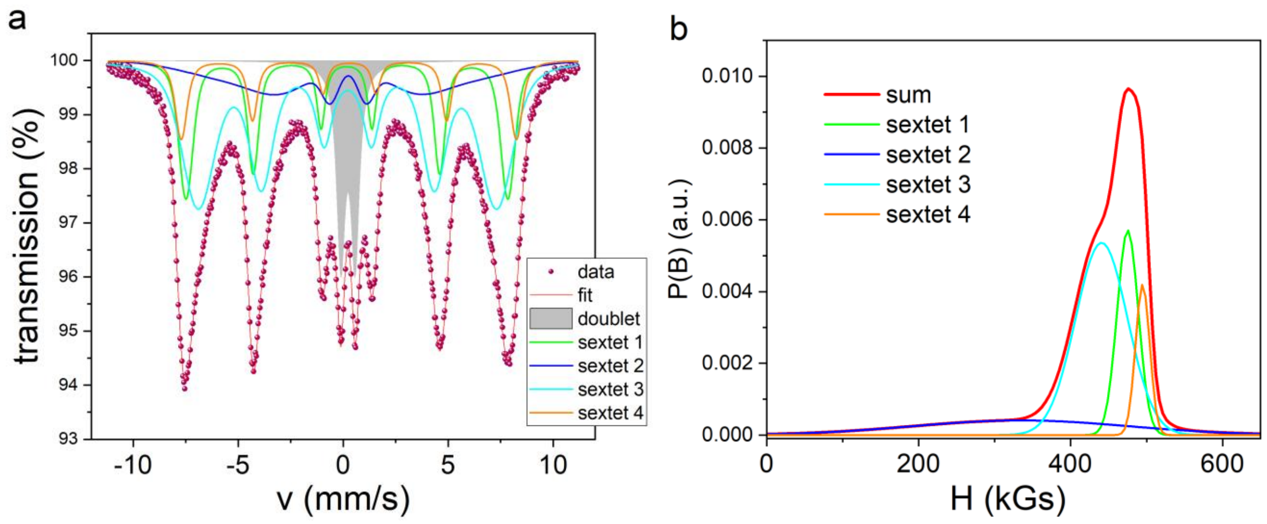

Figure S7. (a) The fit of the Mn-3 sample and the (b) corresponding magnetic hyperfine distribution P(B)-curves derived from the spectrum.

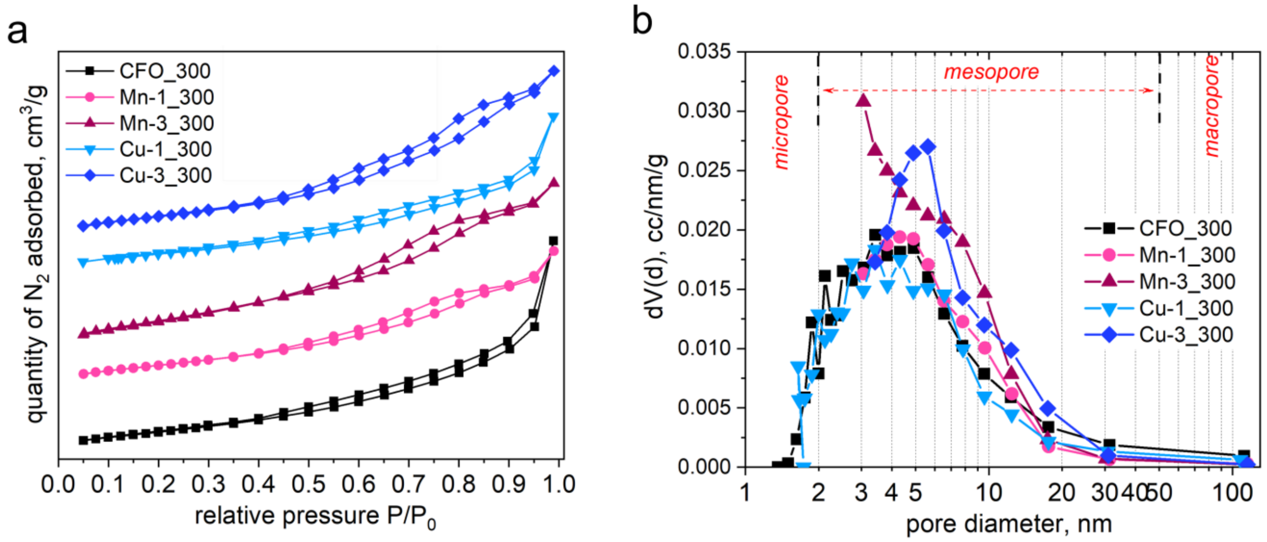

Figure S8. (a) N_2 adsorption/desorption isotherms and (b) pore size distributions for ferrite samples annealed at 300°C.

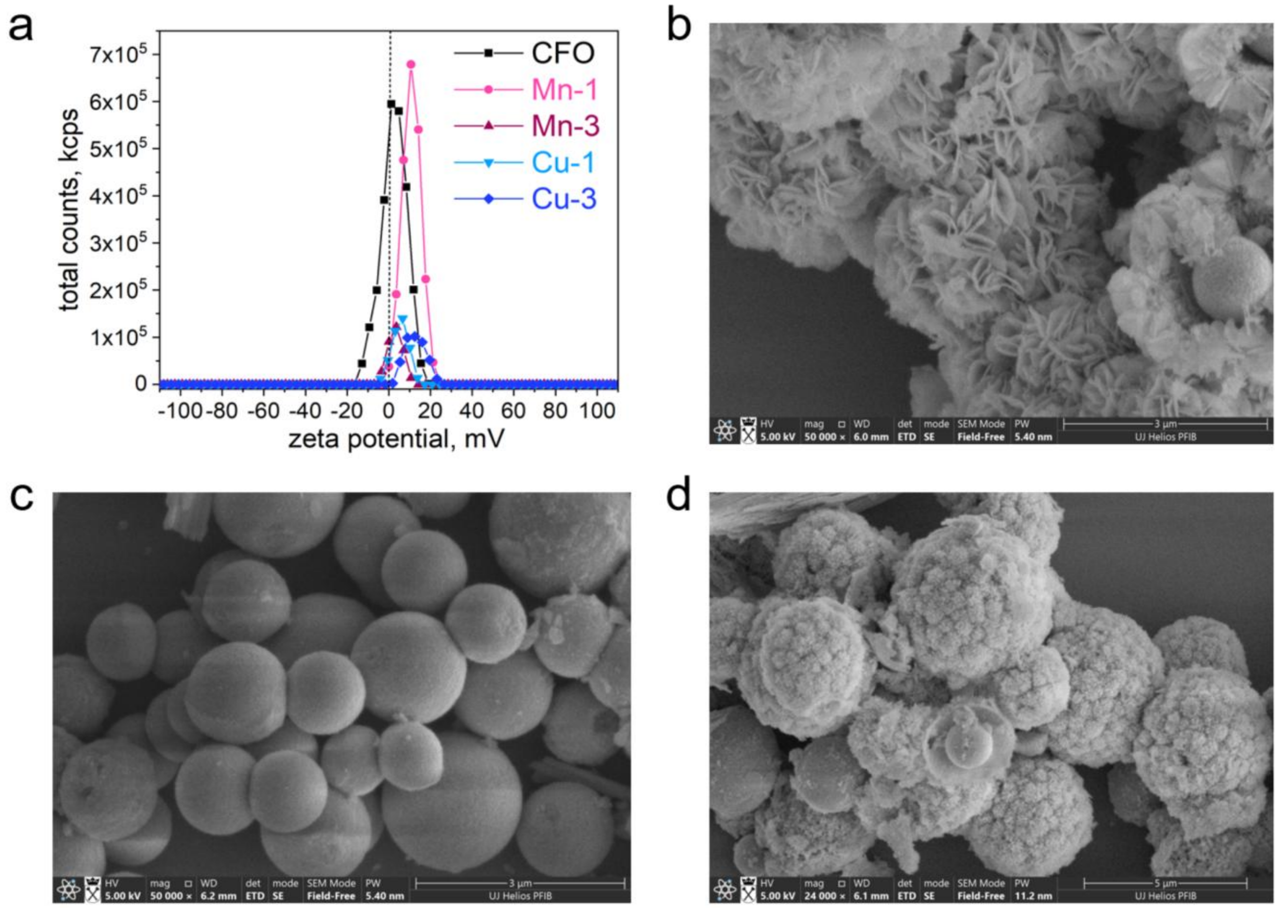

Figure S9. (a) ξ-potential. (b-d) SEM photos of the most representative (b) CFO, (c) Mn-3, and (d) Cu-1 samples.

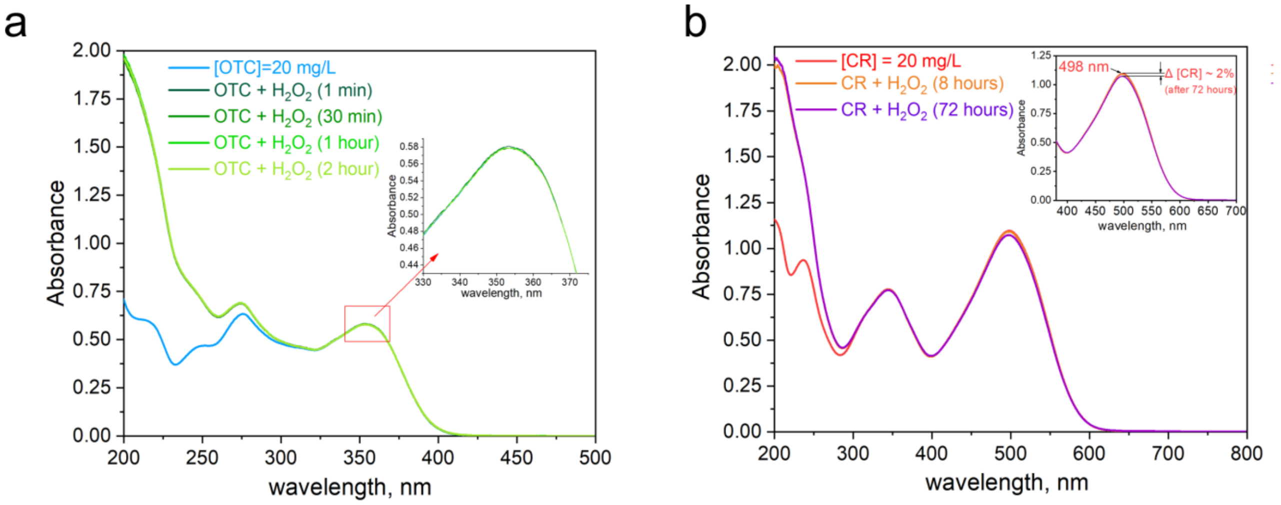

Figure S10. (a) UV-Vis spectra of OTC and OTC+H_2O_2 mixture, taken at 1 min, 30 min, 1 hour, and 2 hours. (b) UV-Vis spectra of CR and CR+H_2O_2 mixture, taken at time intervals of 8 hours and 72 hours.

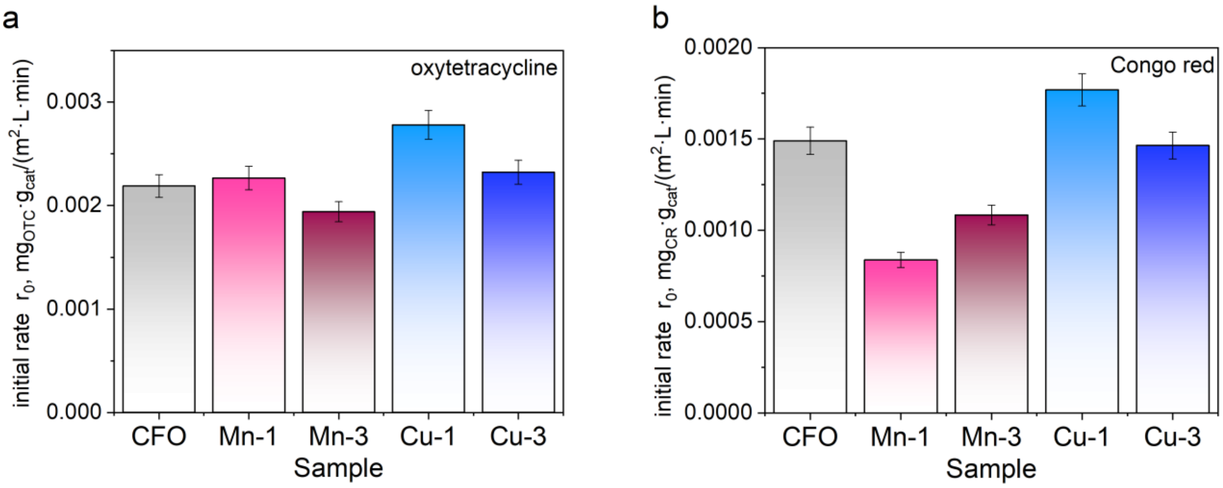

Figure S11. The initial rate of (a) OTC and (b) CR removal normalized to the specific surface area of the samples.

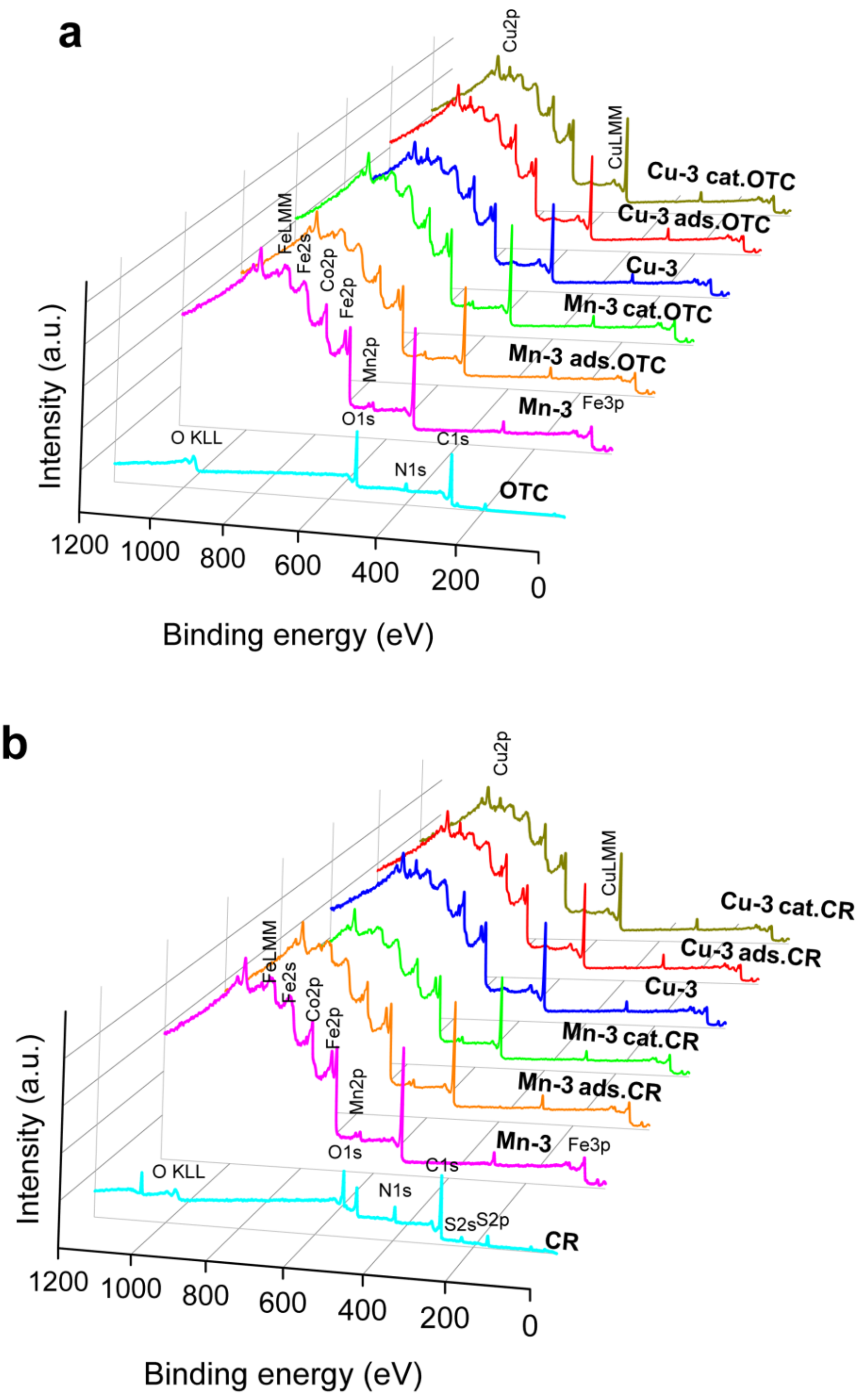

Figure S12. Survey XPS spectra of model OTC (a) and CR (b) pollutants and Mn-3 and Cu-3 ferrites before and after OTC (a) and CR (b) adsorption and catalysis removal.

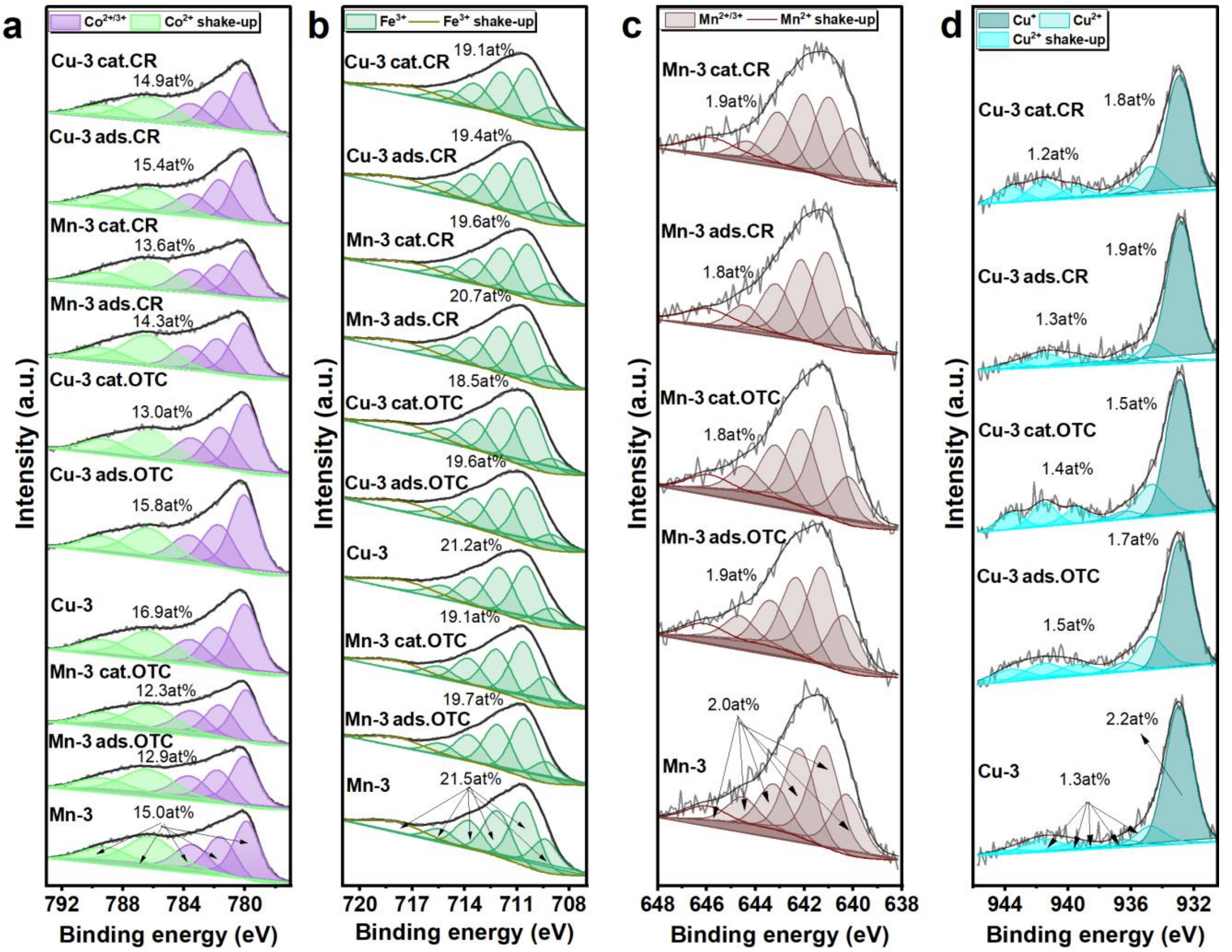

Figure S13. High-resolution XPS spectra of (a) Co $2p_{3/2}$, (b) Fe $2p_{3/2}$, (c) Mn $2p_{3/2}$, and (d) Cu $2p_{3/2}$, for Mn-3 and Cu-3 samples before and after CR/OTC adsorption/catalysis.

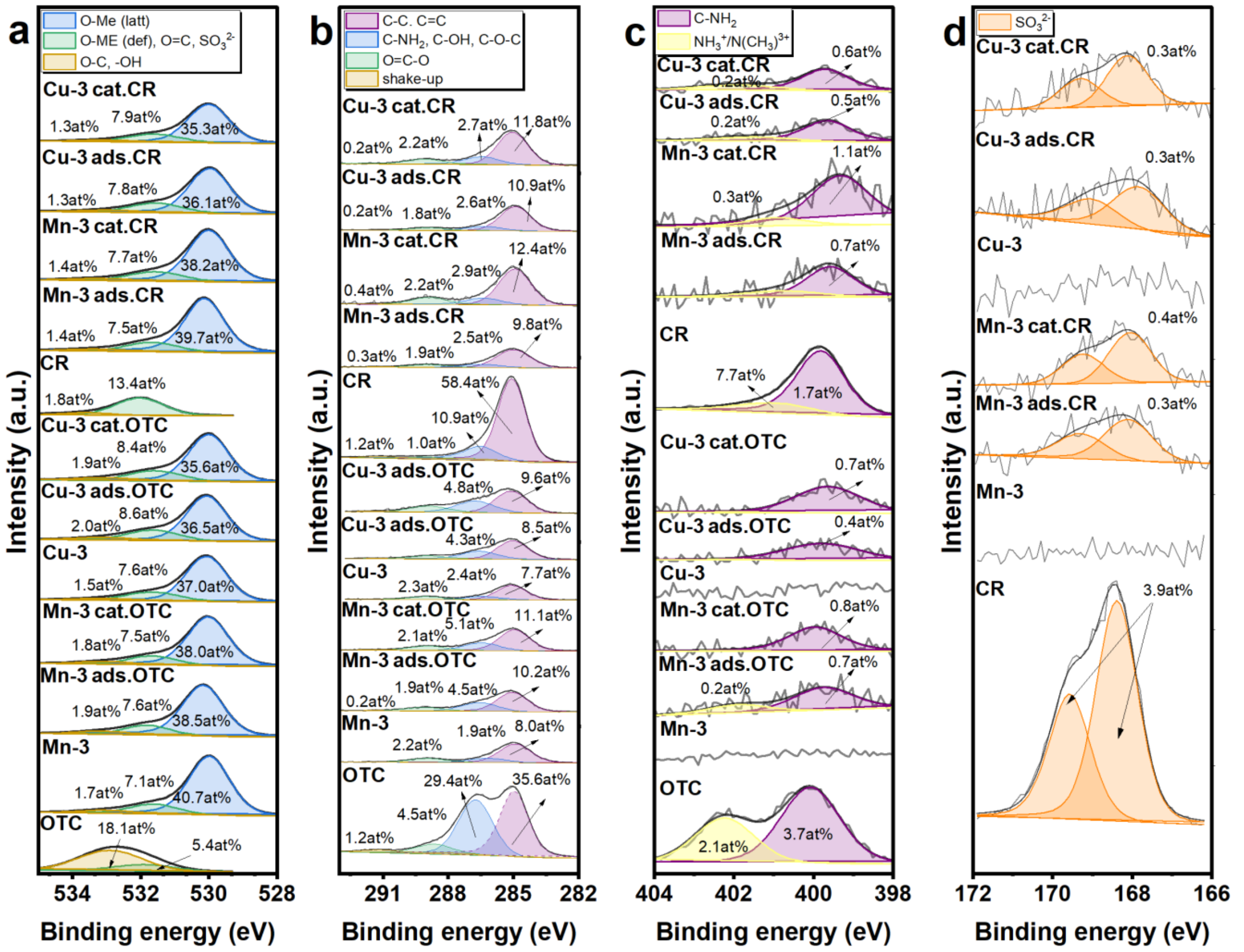

Figure S14. High-resolution XPS spectra of (a) O 1s, (b) C 1s, (c) N 1s, and (d) S 2p for CR dye, OTC powder, and Mn-3, Cu-3 samples before and after CR and OTC adsorption and catalysis.

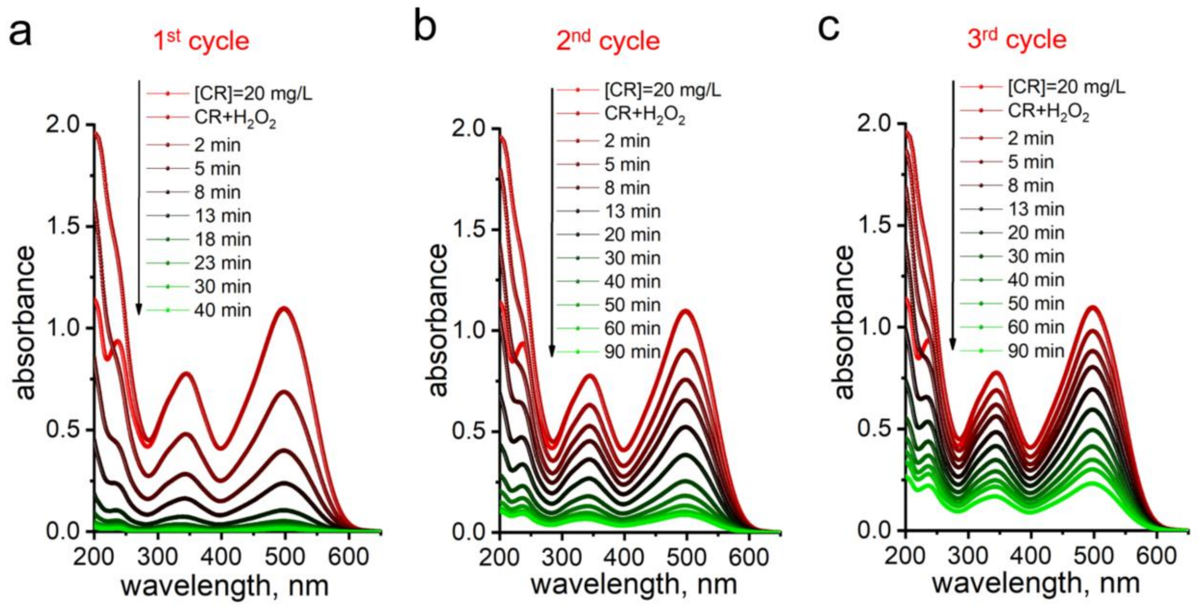

Figure S15. UV-Vis spectra of Congo Red during three consecutive degradation cycles using the Mn-3 sample. Conditions: [CR] = 20 mg/L, V = 25 mL, [Mn-3] = 1 g/L, [H_2O_2]= 10 mM, pH ~ 7, T = 20°C.

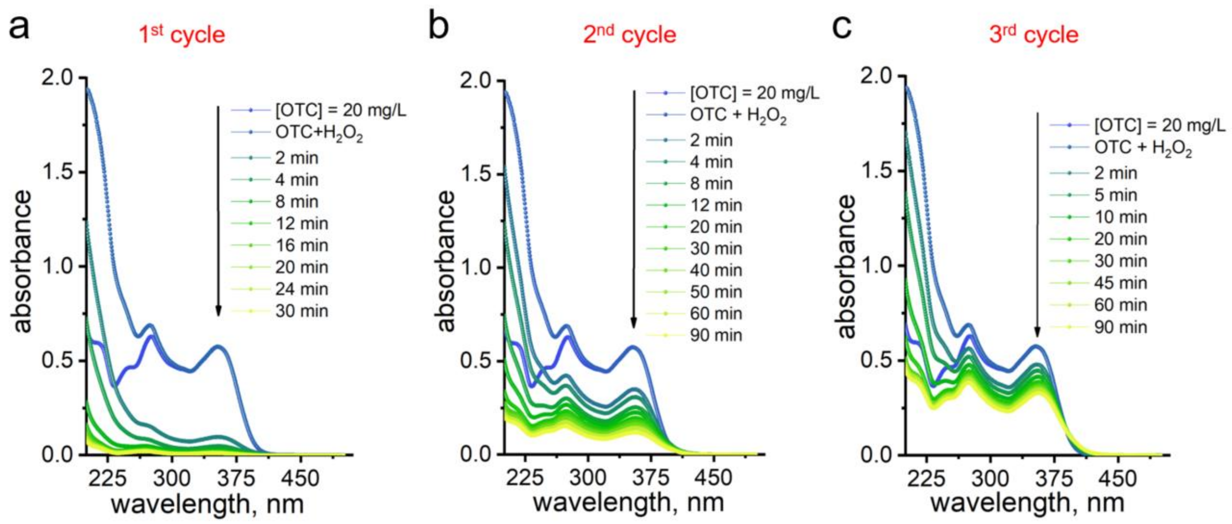

Figure S16. UV-Vis spectra of oxytetracycline during three consecutive degradation cycles using the Mn-3 sample. Conditions: [OTC] = 20 mg/L, V = 25 mL, [Mn-3] = 1 g/L, [H_2O_2]= 10 mM, pH ~ 7, T = 20°C.

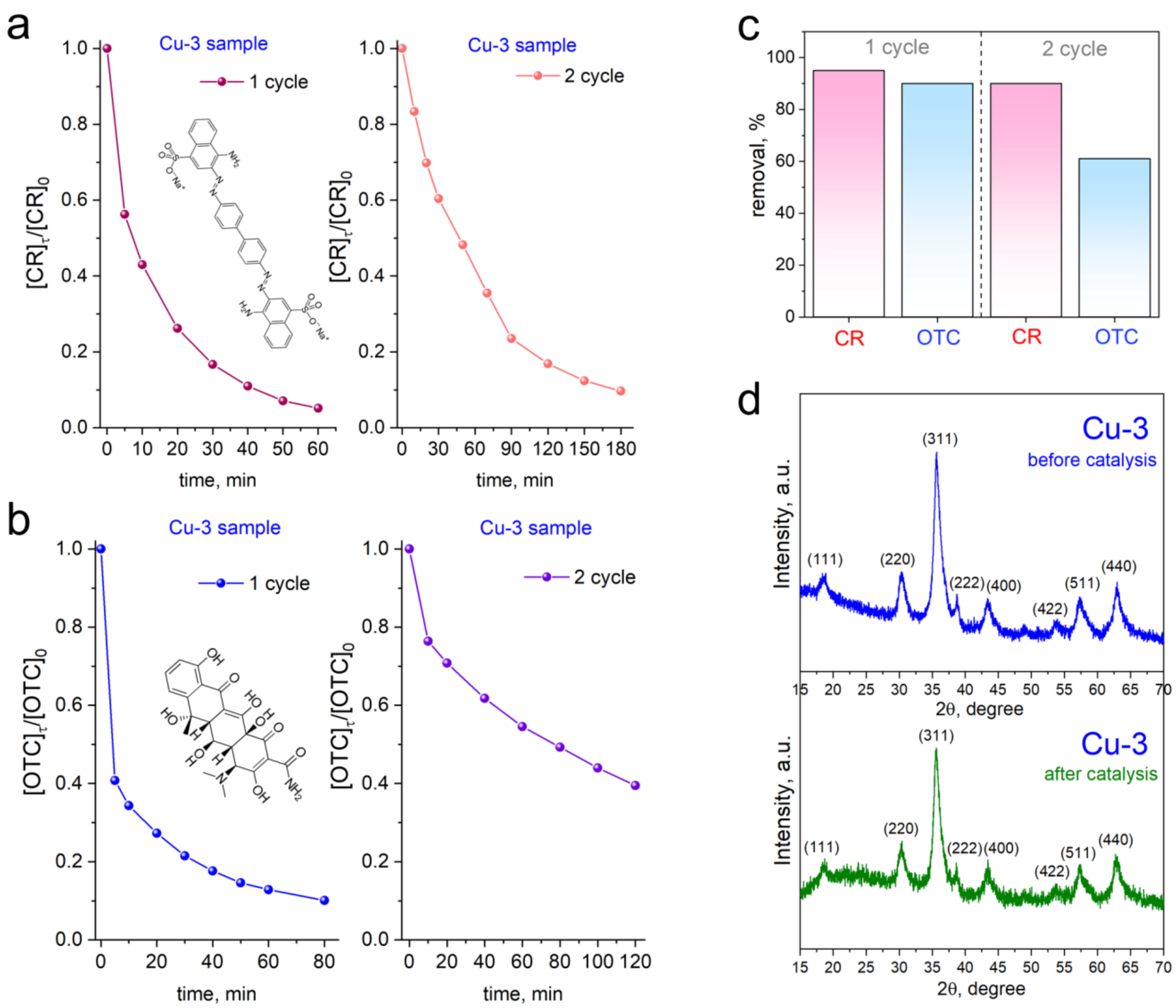

Figure S17. (a,b) Kinetic curves demonstrating the catalytic degradation of the (a) CR and (b) OTC using the Cu-3 sample and H_2O_2 during three cycles. (c) Removal (in %) of CR and OTC during the two cycles. Conditions: [CR] = 20 mg/L, [OTC] = 20 mg/L, V = 25 mL, [catalyst] = 1 g/L, [H_2O_2] = 10 mM, pH ~ 7, T = 20°C. (d) XRD patterns of the Cu-3 samples, obtained before and after removal process.

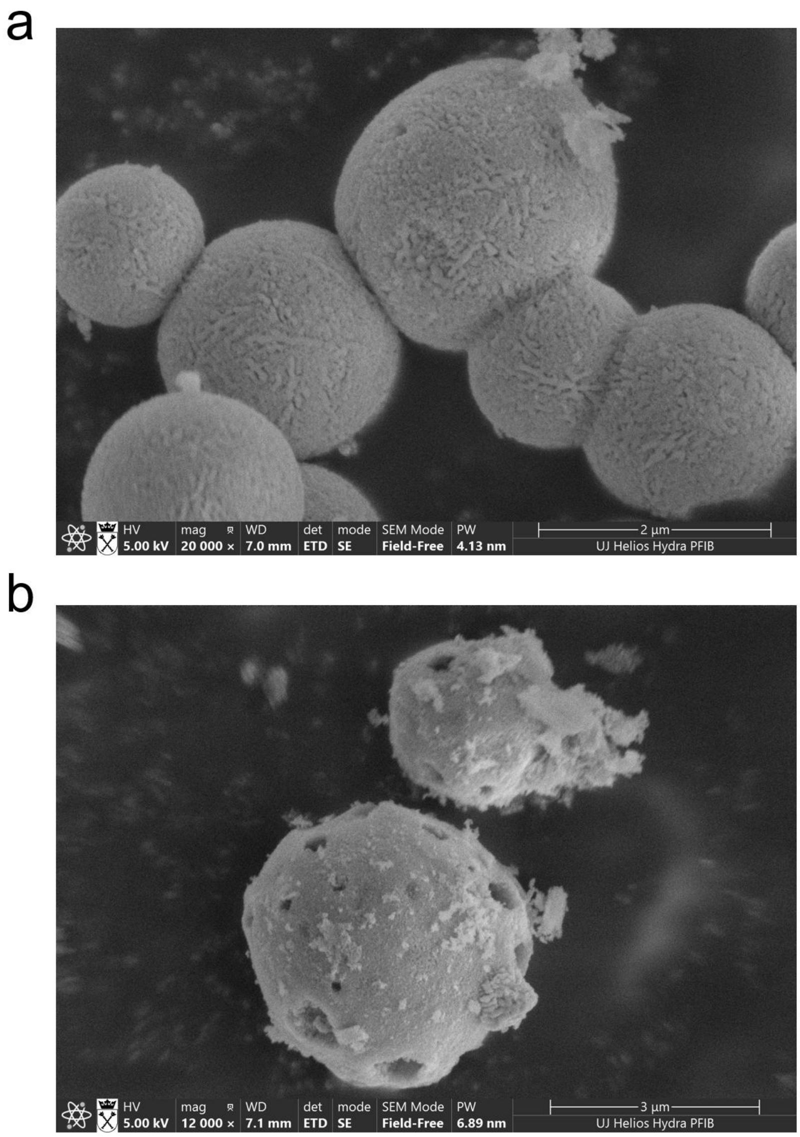

Figure S18. SEM images of (a) Mn-3 and (b) Cu-3 samples after catalysis.

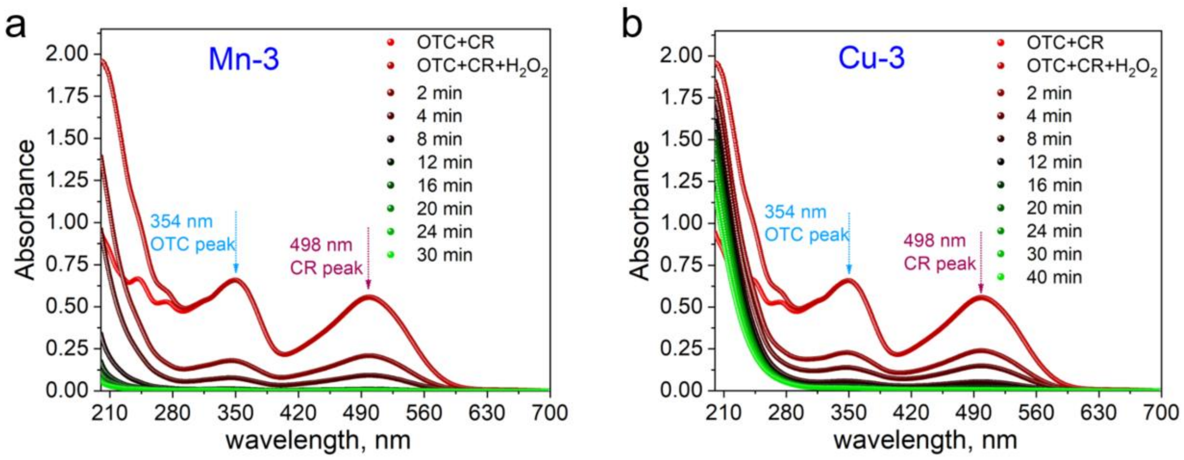

Figure S19. UV-Vis spectra of the mixture of Congo red and oxytetracycline decomposed using (a) Mn-3 and (b) Cu-3 samples. Conditions: [CR] = 10 mg/L, [OTC] = 10 mg/L, V = 25 mL, [catalyst] = 1 g/L, [H_2O_2]= 10 mM, pH ~ 7, T = 20°C.

Table S1. Summary of interplanar spacings and lattice parameters from XRD analysis.

Sample	(hkl)	d, Å	1/d, 1/Å	$1/d^2$, $1/Å^2$	$h^2+k^2+l^2$	$(h^2+k^2+l^2)^{1/2}$	*a*, Å
CFO	111	4.802	0.208247	0.043367	3	1.732051	8.362 ± 0.014
	220	2.96	0.337838	0.114134	8	2.828427	
	311	2.52	0.396825	0.15747	11	3.316625	
	400	2.091	0.47824	0.228714	16	4	
	422	1.701	0.587889	0.345614	24	4.898979	
	511	1.612	0.620347	0.384831	27	5.196152	
	440	1.478	0.67659	0.457774	32	5.656854	
Mn-1	111	4.7903	0.208755	0.043579	3	1.732051	8.367 ± 0.007
	220	2.9507	0.338903	0.114855	8	2.828427	
	311	2.5154	0.397551	0.158047	11	3.316625	
	400	2.0896	0.47856	0.22902	16	4	
	422	1.7031	0.587165	0.344762	24	4.898979	
	511	1.6087	0.62162	0.386411	27	5.196152	
	440	1.479	0.676133	0.457155	32	5.656854	
Mn-3	111	4.8213	0.207413	0.04302	3	1.732051	8.345 ± 0.011
	220	2.9431	0.339778	0.115449	8	2.828427	
	311	2.5181	0.397125	0.157708	11	3.316625	
	400	2.0878	0.478973	0.229415	16	4	
	422	1.6995	0.588408	0.346224	24	4.898979	
	511	1.6087	0.62162	0.386411	27	5.196152	
	440	1.4747	0.678104	0.459825	32	5.656854	
Cu-1	111	4.7903	0.208755	0.043579	3	1.732051	8.371 ± 0.003
	220	2.9507	0.338903	0.114855	8	2.828427	
	311	2.5181	0.397125	0.157708	11	3.316625	
	400	2.0878	0.478973	0.229415	16	4	
	422	1.7067	0.585926	0.343309	24	4.898979	
	511	1.6087	0.62162	0.386411	27	5.196152	
	440	1.479	0.676133	0.457155	32	5.656854	
Cu-3	111	4.8422	0.206518	0.04265	3	1.732051	8.359 ± 0.010
	220	2.9479	0.339225	0.115073	8	2.828427	
	311	2.5165	0.397377	0.157909	11	3.316625	
	400	2.0865	0.479272	0.229701	16	4	
	422	1.7016	0.587682	0.34537	24	4.898979	
	511	1.6102	0.621041	0.385692	27	5.196152	
	440	1.4776	0.676773	0.458022	32	5.656854	

Table S2. Textural characteristics of Mn- and Cu-containing cobalt ferrites, annealed at 300°C.

Adsorbent	S_{BET} $m^2 \cdot g^{-1}$	S_{meso} $m^2 \cdot g^{-1}$	V_p $cm^3 \cdot g^{-1}$	V_{meso} $cm^3 \cdot g^{-1}$	Average pore diameter, nm
CFO_300	100.2	100.2	0.3240	0.3240	13
Mn-1_300	110.1	110.1	0.2153	0.2153	8
Mn-3_300	159.8	159.8	0.2693	0.2693	7
Cu-1_300	98.9	98.9	0.2452	0.2452	10
Cu-3_300	118.4	118.4	0.2652	0.2652	9

Note. S_{meso} and V_{meso} correspond to pore diameters 2 nm < R < 50 nm, respectively.

Table S3. Textural characteristics of Mn- and Cu-containing cobalt ferrites, annealed at 400°C.

Adsorbent	S_{BET} $m^2 \cdot g^{-1}$	S_{meso} $m^2 \cdot g^{-1}$	V_p $cm^3 \cdot g^{-1}$	V_{meso} $cm^3 \cdot g^{-1}$	Average pore diameter, nm
CFO	58.9	58.9	0.2631	0.2631	18
Mn-1	80.7	80.7	0.2040	0.2040	10
Mn-3	115.1	115.1	0.2604	0.2604	9
Cu-1	49.5	49.5	0.2519	0.2519	20
Cu-3	75.4	75.4	0.2434	0.2434	13

Note. S_{meso} and V_{meso} correspond to pore diameters 2 nm < R < 50 nm, respectively.

Table S4. Hydrodynamic size of particles, polydispersity index (PDI), and surface charge of Mn- and Cu-containing ferrites annealed at 400°C.

Sample	Z-average, nm	PDI	Size range, nm	Peak position, nm	ξ, mV
CFO	388.4	0.4677	93–769	330.7	1.943
Mn-1	372.6	0.494	80–660	281.7	10.9
Mn-3	3016	0.1552	1406–2571	1848	3.168
Cu-1	1029	0.4098	146–311<br>769–3477	216.1 (12.45%)<br>1710 (87.55 %)	5.379
Cu-3	802.4	0.5546	80–108<br>361–769	92.22 (12.44 %)<br>539 (87.56 %)	12.73

References

[1] D. Guo, X. Song, F. Li, L. Tan, H. Ma, L. Zhang, Y. Zhao, Oriented synthesis of Co_3O_4 core-shell microspheres for high-performance asymmetric supercapacitor, Colloids Surfaces A Physicochem. Eng. Asp. 546 (2018) 1–8. https://doi.org/https://doi.org/10.1016/j.colsurfa.2018.02.072.

[2] D. Larcher, G. Sudant, R. Patrice, J.-M. Tarascon, Some Insights on the Use of Polyols-Based Metal Alkoxides Powders as Precursors for Tailored Metal-Oxides Particles, Chem. Mater. 15 (2003) 3543–3551. https://doi.org/10.1021/cm030048m.

[3] Y. Zhang, Q. Yang, J. Liang, Y. Luo, Q. Liu, Y. Yang, X. Sun, Fe-glycerate microspheres as a heterogeneous catalyst to activate peroxymonosulfate for efficient degradation of methylene blue, J. Phys. Chem. Solids. 169 (2022) 110893. https://doi.org/https://doi.org/10.1016/j.jpcs.2022.110893.

[4] B. Dey, M. Bououdina, G.A. elfadeel, P. Dhamodharan, S. AsathBahadur, M. Venkateshwarlu, C. Manoharan, Tuning the gas sensing properties of spinel ferrite $NiFe_2O_4$ nanoparticles by Cu doping, J. Alloys Compd. 970 (2024) 172711. https://doi.org/https://doi.org/10.1016/j.jallcom.2023.172711.

[5] Y.V. Kaneti, R.R. Salunkhe, N.L. Wulan Septiani, C. Young, X. Jiang, Y.-B. He, Y.-M. Kang, Y. Sugahara, Y. Yamauchi, General template-free strategy for fabricating mesoporous two-dimensional mixed oxide nanosheets via self-deconstruction/reconstruction of monodispersed metal glycerate nanospheres, J. Mater. Chem. A. 6 (2018) 5971–5983. https://doi.org/10.1039/C8TA00008E.

[6] K. Patil, K. Jangam, S. Patange, S. Balgude, A.G.G. Al-Sehemi, H. Pawar, P. More, Influence of Cu–Mg substituted $ZnFe_2O_4$ ferrite as a highly efficient nanocatalyst for dye degradation and 4-nitrophenol reduction, J. Phys. Chem. Solids. 167 (2022) 110783. https://doi.org/https://doi.org/10.1016/j.jpcs.2022.110783.

[7] M. Moradi, F. Hasanvandian, M. Ghahraman Afshar, A. Larimi, F. Khorasheh, E. Niknam, S. Rahman Setayesh, Incorporation of Fe in mixed CoCu-alkoxide hollow sphere for enhancing the electrochemical water oxidation performance, Mater. Today Chem. 22 (2021) 100586. https://doi.org/https://doi.org/10.1016/j.mtchem.2021.100586.

[8] T. Tatarchuk, I. Starko, Mesoporous La-substituted nickel-cobalt ferrites synthesized via reduction method resulting in significantly enhanced adsorption properties, J. Environ. Chem. Eng. (2025) 115657. https://doi.org/https://doi.org/10.1016/j.jece.2025.115657.

[9] T. Tatarchuk, V. Bilovol, A. Shyichuk, I. Danyliuk, K. Sokołowski, M. Gajewska, Mesoporous Co-Mn ferrites as highly radical-forming catalysts for wet peroxide oxidation of 4-nitrophenol, Appl. Surf. Sci. 690 (2025) 162610.

https://doi.org/https://doi.org/10.1016/j.apsusc.2025.162610.

[10] T.R. Tatarchuk, N.D. Paliychuk, M. Bououdina, B. Al-Najar, M. Pacia, W. Macyk, A. Shyichuk, Effect of cobalt substitution on structural, elastic, magnetic and optical properties of zinc ferrite nanoparticles, J. Alloys Compd. 731 (2018) 1256–1266. https://doi.org/10.1016/j.jallcom.2017.10.103.

[11] Y. Ren, Z. Ma, R.E.E. Morris, Z. Liu, F. Jiao, S. Dai, P.G.G. Bruce, A solid with a hierarchical tetramodal micro-meso-macro pore size distribution, Nat. Commun. 4 (2013) 2015. https://doi.org/10.1038/ncomms3015.
